# Fluorination of alkoxide ligands: neither too much, nor too little for optimal ligand field strength

**DOI:** 10.1039/d6sc05403j

**Published:** 2026-08-19

**Authors:** Felix J. de Zwart, Noah Gehr, Sophie W. Anferov, Joël Landis, Christophe Copéret

**Affiliations:** a Department of Chemistry and Applied Biosciences ETH Zürich Zürich CH-8093 Switzerland ccoperet@ethz.ch

## Abstract

Fluorination is generally expected to act predictably in ligand design: each added fluorine atom withdraws electron density, weakens σ-donation, and steadily modulates reactivity. However, reported catalytic systems show activity instead peaking at intermediate fluorination—a non-monotonic trend that simple donor-strength arguments cannot explain. To disentangle this non-monotonicity, we prepared a series of homoleptic Mo(v) alkoxides bearing fluorinated *tert*-butoxide ligands *via* Mo(iv) → Mo(v/iii) disproportionation. As oxo-free d^1^ species with trigonal-bipyramidal geometry, these complexes provide a direct EPR handle on ligand-field splitting, indicating strengthened axial fields at partial fluorination. Computations reveal enhanced donor–acceptor overlap and favorable electrostatics at intermediate F-content, rationalizing the fluorination sweet spot observed experimentally in catalysis. ^17^O NMR calculations pinpoint the physical origin of this effect towards C–CF_3_ σ* acceptor orbitals. Notably, computational exploration of Schrock-type catalysts, for which the influence of fluoroalkoxide ligands is well-known, shows that the specific alkoxide orientation can have a net influence of up to 6 kcal mol^−1^ on the ground and transition state energies for the metallacyclobutane formation during the olefin metathesis process. The findings provide an understanding of fluorination and can be used to develop new alkoxide ligands and analogous promising synthons for alkoxide-containing catalysts.

## Abstract 2

A fluorination sweet spot emerges in Mo(v) alkoxides: partial CF_3_ substitution strengthens the ligand field. EPR, energy decomposition, and ^17^O NMR calculations reveal that C–CF_3_ σ* acceptor orbitals tune Mo–O bonding and catalyst conformations.

## Graphical abstract

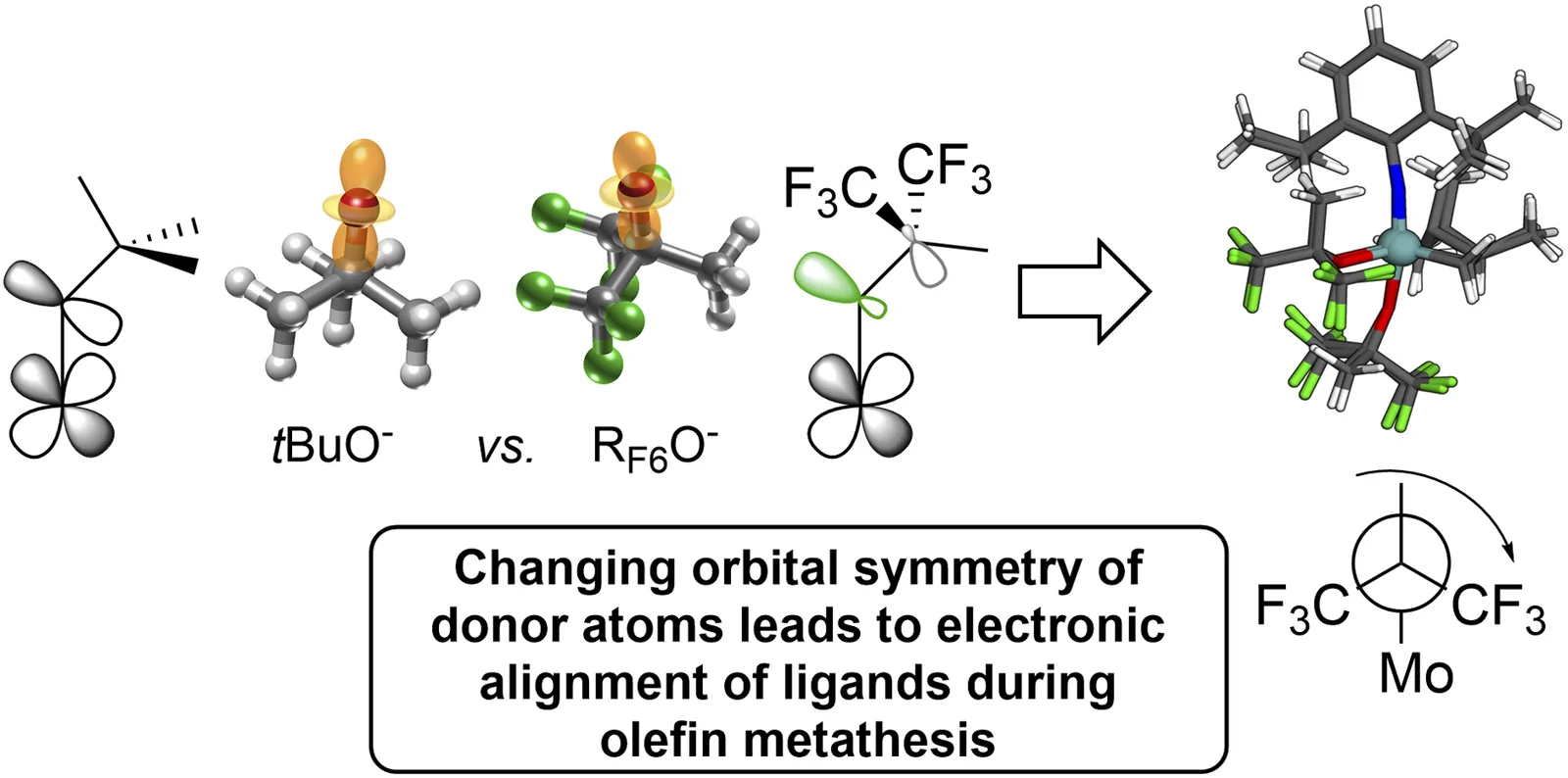

## Introduction

**Fig. 1:**
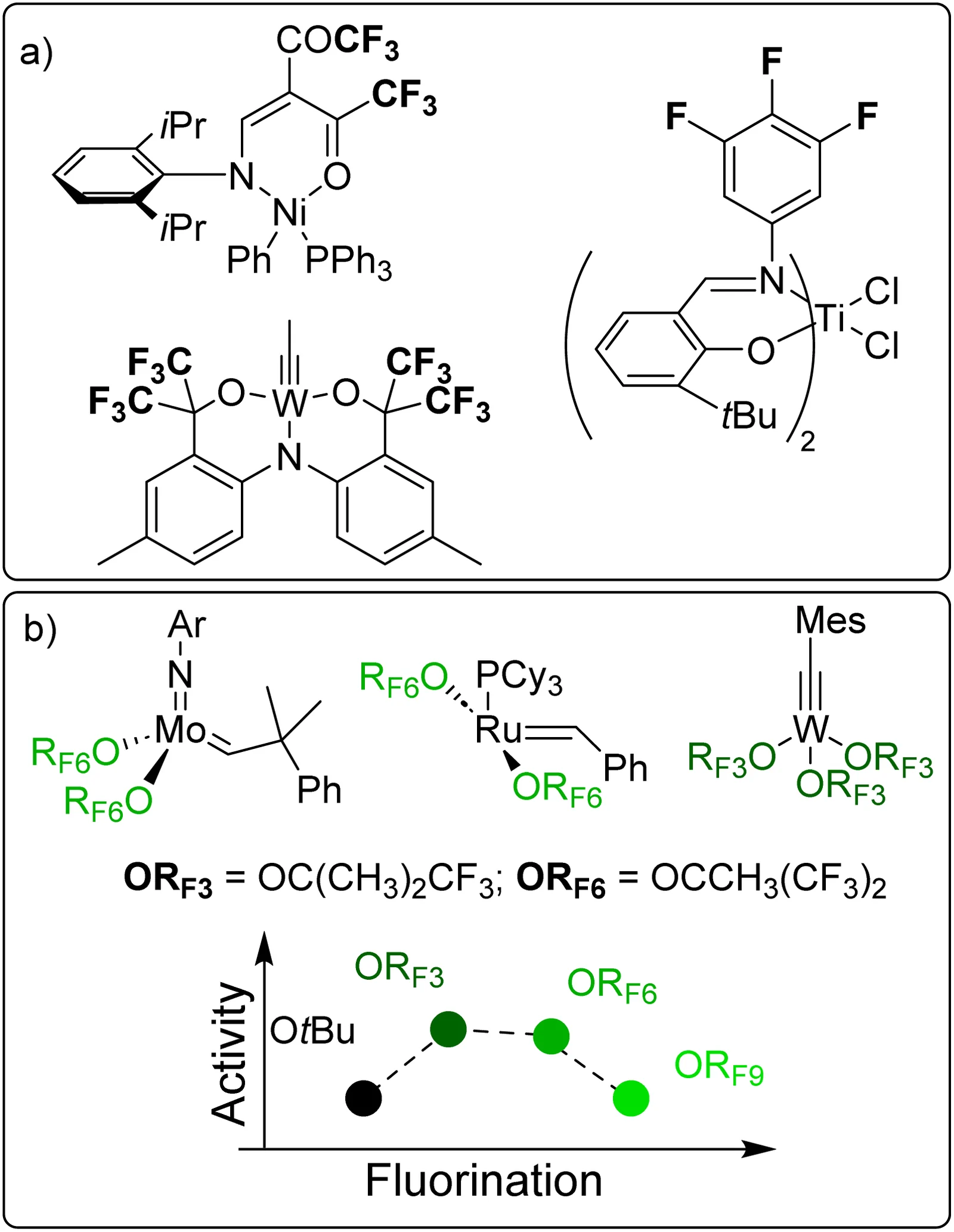
(a) Selected examples of catalysts with intermediate fluorine substitution.^5–20^ (b) Fluorination weakens donation, yet catalytic activity sometimes peaks at intermediate substitution in metathesis.

Fluorination is widely used in ligand design to modulate the electronic and steric properties of transition-metal complexes.^1,2^ The consequences are generally considered relatively straightforward: substituting hydrogen with fluorine atoms withdraws electron density from the donor atom, weakening σ-donation to the metal center in a monotonic trend.^3,4^ This associated positive charge and induced electrophilicity of the metal center directly affect catalytic performances.^5,6^ Notable examples include ethylene polymerization catalysts based on Ni (Fig. 1a)^7^ as well as W pincer complexes, where the presence of fluorine in the ligand modifies the activity of metal sites.^8,9^ In addition, fluorine-incorporation can also induce supramolecular effects in the second coordination sphere, such as hydrogen bonding to substrates, as is the case with the FI catalysts based on Ti (Fig. 1a).^10,11^ While one could expect a monotonous behavior as a function of the number of fluorine atoms in the ligand, catalytic performances often peak at intermediate levels of fluorination. This is probably best illustrated across Schrock-type Mo- and W-based alkene and alkyne metathesis catalysts, with both non-fluorinated and perfluorinated alkoxide ligands often exhibiting lower reaction rates (Fig. 1b).^12–18^

Similar optima have also been observed for the alkoxide-based Grubbs-type alkene metathesis catalysts as well as the most recent Fe-based systems, suggesting that the underlying effects are intrinsic to alkoxides and not metal specific.^19,20^ The counterintuitive trend, in which catalytic activity peaks at intermediate fluorination, probably indicates that fluorination influences more than just σ-donor strength.^21^

To investigate this phenomenon, we reasoned that a molecular platform is required where the impact of alkoxy ligand fluorination on the electronic structure (ligand field/orbital splitting) of the metal centers can be directly and spectroscopically evaluated. We therefore focus our study on a series of well-defined homoleptic molecular compounds with a d^1^ configuration, having five alkoxide ligands with various degrees of fluorination, namely Mo^V^(O^*t*^Bu_Fi_)_5_ (O^*t*^Bu_Fi_ with *i* = 0, 3, 6 and 9), considering the recently optimized synthesis of the F_0_ derivative.^22^ The specific d^1^ electronic configuration is ideally poised for probing the electronic structure from EPR spectroscopy.^23^ Notably, the *g*-tensor anisotropy provides a direct reflection of the ligand field splitting, allowing us to quantify how fluorination modulates the electronic structure.^24,25^ Here, the detailed experimental and computational study of fluorinated Mo(v) alkoxide complexes enable us to show how electrostatics, covalency, and orbital orientation together shape their electronic structures, and, more strikingly, how intermediate fluorination achieves an optimal balance among these factors.

## Results and discussion

### Synthesis and structures of homoleptic Mo(v) alkoxides

**Scheme 1:**
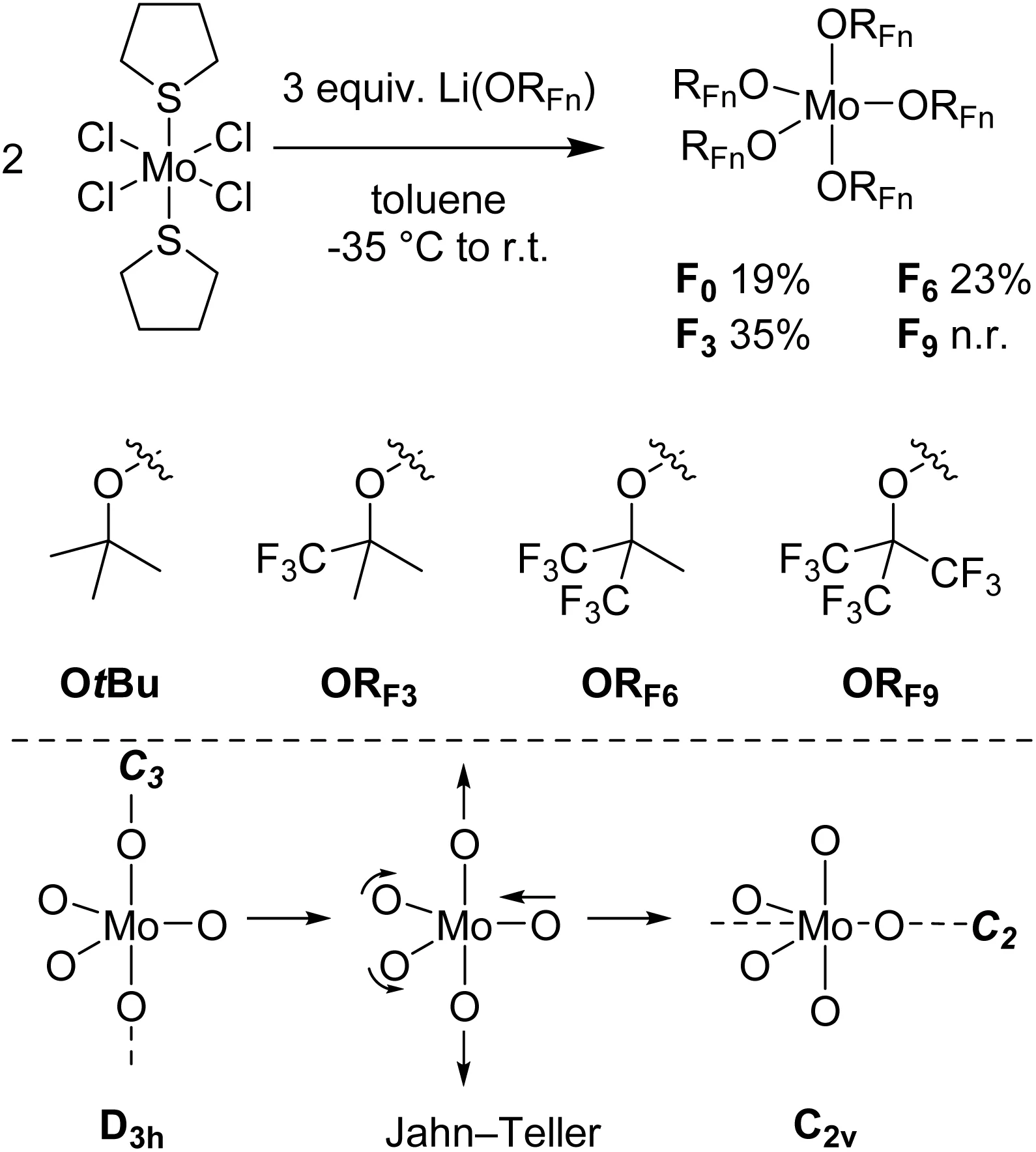
(a) Synthesis of homoleptic Mo(v) alkoxides through disproportionation of Mo(iv). (b) Lowering of symmetry through Jahn–Teller distortion from *D*_3h_ to *C*_2v_.

We first prepared a series of Mo^V^(OR_Fn_)_5_ compounds by reacting LiOR_Fn_ with MoCl_4_(tht)_2_.^22^ We find that, for this reaction, the tetrathiophene (tht) precursor is superior to the tetrahydrofuran (thf)^26^ analog, MoCl_4_(thf)_2_, yielding more consistently reproducible yields across this series. Note that the formation of these compounds likely involves complex reaction pathways including disproportionation steps, limiting the theoretical yield to 50%, as Mo is acting as the terminal oxidant in the reaction. Mo(O^*t*^Bu)_5_ is generated in a 19% yield after sublimation. Treatment of MoCl_4_(tht)_2_ with LiO^*t*^Bu-F_3_ and LiO^*t*^Bu-F_6_ under identical conditions furnishes the corresponding homoleptic Mo(v) complexes in pure form after crystallization from pentane in 35% and 23% yields, respectively (Scheme 1). Notably, no conversion is observed in the case of LiO^*t*^Bu-F_9_. In addition, while Mo(O^*t*^Bu)_5_ is thermally sensitive and difficult to purify *via* sublimation, the OR_F3_ and OR_F6_ congeners are considerably more stable and easier to handle and obtain.

Single crystals of Mo(OR_F3_)_5_ and Mo(OR_F6_)_5_ suitable for X-ray diffraction (XRD) are grown from pentane at −35 °C. Mo(OR_F3_)_5_ crystallizes in the monoclinic space group *P*2_1_/*c*, and Mo(OR_F6_)_5_ in the orthorhombic space group *Pca*2_1_. A comparison of the powder XRD pattern of the acquired crystals for Mo(OR_F3_)_5_ and the simulated pattern from single-crystal XRD confirms that the bulk consists of this compound (Fig. S1), with no presence of Mo_2_(OR_F3_)_6_, as was observed for the parent Mo(O^*t*^Bu)_5_.

**Fig. 2:**
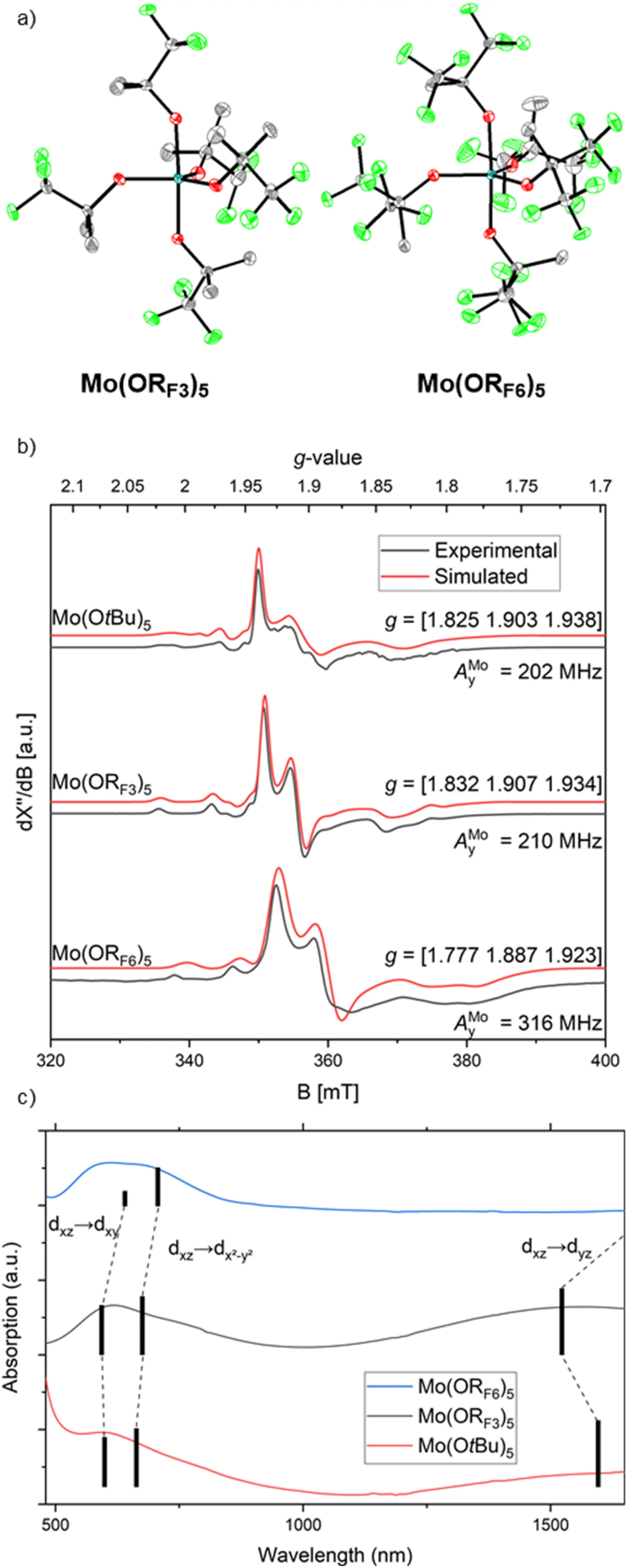
(a) ORTEP plots (50% probability, hydrogen atoms omitted for clarity) of Mo(OR_F3_)_5_ (left) and Mo(OR_F6_)_5_ (right). (b) X-band EPR spectra in toluene glass and (c) UV-vis-NIR spectra measured in pentane at 10 mM, and TD-DFT calculated d–d transitions as sticks at the B3LYP/x2c-TZVP level of theory.

Single-crystal XRD confirms that both Mo(OR_F3_)_5_ and Mo(OR_F6_)_5_ adopt a distorted TBP geometry, as illustrated by the ORTEP plots (50% probability; H omitted) in Fig. 2. The reported crystal structure for Mo(O^*t*^Bu)_5_ can be similarly described as an approximately trigonal bipyramidal structure, with notable distortions.^22^ In this d^1^ system, the distortion, which reduces the symmetry from *D*_3h_ to *C*_2v_, is due to the Jahn–Teller effect and driven by the SOMO, which lifts the degeneracy of the *e*″ set (Scheme 1). The equatorial plane contains one shortened bond due to the lifted degeneracy of the *e*″ orbital set (F_0_, Mo–O_eq_: 1.858, 1.870, 1.870 Å; F_3_, Mo–O_eq_: 1.828, 1.881, 1.895 Å; F_6_, Mo–O_eq_: 1.825, 1.895, 1.909 Å). The Mo–O_ax_ bond distance is considerably longer and increases with fluorination from 1.924 (F_0_) to 1.927 (F_3_) to 1.930 Å (F_6_).

Relative to the non-fluorinated parent compound, the fluorinated congeners are less distorted TBP according to the Addison metric: *τ*_5_ increases from 0.74 in Mo(O^*t*^Bu)_5_ to 0.87 (F_3_) and 0.86 (F_6_), where *τ*_5_ = 1 for an ideal TBP structure (Table 1).^27^ This is reflected in the 
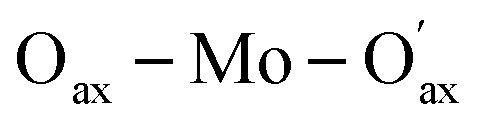
 angle changing from 169.1 (F_0_) to 172.9 (F_3_) to 171.8° (F_6_). Notably, in Mo(OR_F3_)_5_, all CF_3_ moieties are oriented in an antiperiplanar fashion to molybdenum (avg. C_CF_3__–C–O–Mo 165°). Finding this alignment for all OR_F3_ ligands in the crystal structure is surprising, considering that it is the spatially the least favored, suggesting some underlying electronic effects (*vide infra*). In the hexafluoro congener Mo(OR_F6_)_5_, the CH_3_ moieties are gauche to molybdenum (avg. C_CH_3__–C–O–Mo 31°), which aligns the space between both CF_3_ groups as anti to molybdenum. Full metrical parameters and thermal ellipsoids are provided in the SI.

**Table 1:** Experimental spectroscopic features of homoleptic Mo(v) alkoxides

	Mo(O^*t*^Bu)_5_	Mo(OR_F3_)_5_	Mo(OR_F6_)_5_
*τ*_5_	0.74	0.87	0.86
*δ*_H_ (ppm)	7.43	13.06	19.23
*A* ^Mo^ (MHz)	202	210	316
*g*_*x*_	1.938	1.934	1.923
*g*_*y*_	1.903	1.907	1.887
*g*_*z*_	1.825	1.832	1.777

### Spectroscopic trends across the fluorination series

We then compare the three synthetically accessible homoleptic Mo(v) compounds by various techniques. First, the UV/vis spectrum of the parent Mo(O^*t*^Bu)_5_ in pentane displays a broad absorption maximum at 592 nm and a NIR band outside the solvent window of 1650 nm (Fig. 2c), in line with reported MCD bands at 595 and 1685 nm.^22^ The fluorinated complex Mo(OR_F3_)_5_ has a red-shifted maximum at 618 nm in the visible range, and a blue-shifted NIR band centered at 1561 nm. The broad NIR band can be tentatively assigned to a Jahn–Teller-induced *e*″ → *e*″ transition, confirming the lowering of symmetry in solution. This band is not observed for Mo(OR_F6_)_5_, which has a clearly bimodal peak at 602 and 692 nm (*vide infra* for discussion on the assignment). Second, the paramagnetic ^1^H-NMR displays a single broad feature, corresponding to the methyl groups of the alkoxide ligand, in all cases that shifts downfield with increasing fluorination (Table 1), with a chemical shift varying from 7.43 to 13.06 to 19.23 ppm for the F_0_, F_3_ to F_6_ compounds, respectively. Third, the X-band EPR spectra of Mo(OR_F3_)_5_ and Mo(OR_F6_)_5_ display well-resolved signals characteristic of an *S* = 1/2 Mo(v) center with rhombic symmetry (Fig. 2b), with spectral patterns closely resembling that of the parent Mo(O^*t*^Bu)_5_ complex. In particular, both complexes display two distinct signals around *g* = 1.9, and one at higher field around *g* = 1.8. Furthermore, a resolved ^95^Mo (*I* = 5/2) hyperfine coupling to the central *g*-component is observed, indicating a localization of spin density on Mo. These EPR signatures are consistent with a d^1^ center in a distorted trigonal–bipyramidal ligand field. Simulation of the spectra provided a satisfactory fit with a rhombic *g*-tensor and anisotropic broadening. The *g*-tensor components extracted from the simulation show a linear decrease in the low field *g*_*x*_ component, the *g*_*y*_ and *g*_*z*_ components first increase, from Mo(O^*t*^Bu)_5_ to Mo(OR_F3_)_5_, before decreasing again in Mo(OR_F6_)_5_ (Table 1). Compared to reference Mo^V^ complexes, the F_6_ complex has an unusually low *g*_*z*_ value of 1.777.^28^ Parallel to the *g*-value trends, the resolved component of the ^95^Mo hyperfine coupling tensor rises from 202 MHz in Mo(O^*t*^Bu)_5_ to 210 MHz in Mo(OR_F3_)_5_ and 316 MHz in Mo(OR_F6_)_5_. Overall, spectroscopically these compounds show both linear trends (*δ*_H_, *g*_*x*,_*A*^Mo^) and non-linear trends (UV/vis, *τ*_5_, *g*_*y*_, *g*_*z*_). To disentangle what the physical origins are behind these signatures, we next carried out an extensive computational study on this series and extended it to Mo(OR_F9_)_5_.

### Relating the EPR *g*-tensor to ligand-field splitting

The gas phase geometries of the molybdenum complexes are first computed using DFT at the B3LYP/def2-TZVPD/D3(BJ) level of theory, with diffuse functions to accurately represent anionic valence orbitals.^29–31^ Optimization starting from the crystal structures provided gas phase geometries with similarly distorted TBP geometries. The putative Mo(OR_F9_)_5_ complex is also included, and it retains the same geometry. In all cases, this only slightly relaxes the hydrogen atoms, all DFT calculated molybdenum–oxygen bonds stay within 0.03 Å of their SC-XRD distances (Table 2, RMSE 0.015 Å). Most importantly, within all complexes the Mo–O_ax_ axial bonds are elongated by 0.05 Å relative to the equatorial bonds, and one of the Mo–O_eq′_ bonds in the equatorial plane remains shortened by 0.06 Å.

**Table 2:** Molybdenum–oxygen bond distances (Å) of homoleptic Mo(v) alkoxides compared to DFT models

Compound		Mo–O_ax_ (Å)	Mo–O_eq_ (Å)	Mo–O_eq′_ (Å)
Mo(O^*t*^Bu)_5_	DFT	1.950, 1.942	1.891, 1.898	1.842
	Exp^a^	1.879(6)	1.870(5)	1.858(10)
Mo(OR_F3_)_5_	DFT	1.952, 1.926	1.905, 1.897	1.832
	Exp	1.940(1), 1.927(1)	1.895(1), 1.881(1)	1.8278(2)
Mo(OR_F6_)_5_	DFT	1.961, 1.934	1.899, 1.887	1.836
	Exp	1.942(4), 1.927(4)	1.893(3), 1.885(4)	1.833(8)
Mo(OR_F9_)_5_	DFT	1.968, 1.957	1.908, 1.878	1.875

a Literature values taken from ref. 22.

**Fig. 3:**
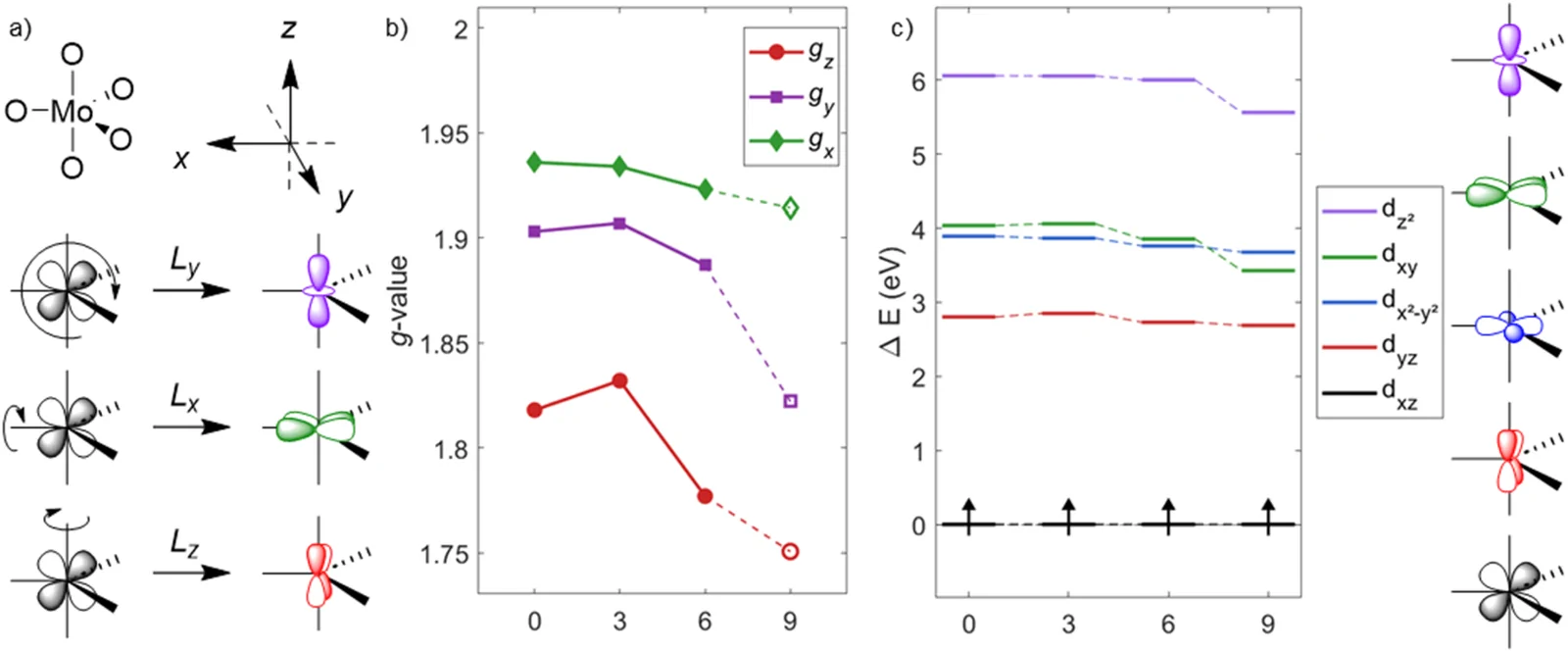
Qualitative orbital analysis of *g*-tensor anisotropy in Mo(v) complexes. (a) Schematic representation of the angular momentum operators acting on the d_*xz*_ SOMO. (b) Experimental (computed for Mo(OR_F9_)_5_) *g*-values as a function of number of fluorines on the *tert*-butoxide scaffold. (c) Calculated energies of the five canonical d-orbitals in Mo(OR_Fn_)_5_ relative to the SOMO.

Electronic properties are next obtained using single point calculations in ORCA on the optimized structures using B3LYP with an all-electron basis set and relativistic corrections implemented by the two-component X2C model and a mean-field spin–orbit operator. TDDFT calculations allowed us to assign the bands observed in the UV-vis spectra shown in Fig. 2. In all three complexes, the first three excited states correspond to >90% pure SOMO–LUMO, SOMO–LUMO+1 and SOMO–LUMO+2 transitions (Table 3). These correspond to d–d transitions within the *e*″ and *e*′ sets of orbitals. No pure *e*″ → a_1_ absorptions are found, as they are Laporte forbidden. The lack of an observable NIR band for Mo(OR_F6_)_5_ is in line with calculations, as it shifts outside the measurement window. Notably, the calculated EPR *g*-tensors are in good (semi-quantitative) agreement with the experimental data (*cf.*Tables 1–3).^32^ DFT consistently overestimates the individual *g*-components by ∼0.02, with the largest deviations in the *g*_*z*_ component, presumably due, at least in part, to the larger experimental error in determining this high field feature. This consistent overestimation allowed us to use linear regression between DFT-calculated and experimental values to compute a corrected *g*-tensor for the putative Mo(OR_F9_)_5_ complex as *g*_*x*,*y*,*z*_ = [1.914, 1.822, 1.751] (details in the SI). Visualizing the calculated *g*-tensor confirms that in all complexes, *g*_*z*_ is lying along the axial ligands, *g*_*x*_ is lying along the longest Mo–O_eq_ bond, and *g*_*y*_ is perpendicular to these components (Fig. 3a). Most importantly, DFT also predicts the linear decrease in *g*_*x*_ with increasing fluorination, and the ordering of *g*_*y*_ and *g*_*z*_ as Mo(OR_F3_)_5_ > Mo(O^*t*^Bu)_5_ > Mo(OR_F6_)_5_ > Mo(OR_F9_)_5_. With computations supporting non monotonic behavior trends in *g*-components, we set out to use qualitative orbital analysis with the EPR *g*-tensor to support this experimentally.

**Table 3:** DFT calculated electronic properties of Mo(OR_Fn_) series. UV/vis transitions (nm) and calculated *g*-tensor

	Mo(O^*t*^Bu)_5_	Mo(OR_F3_)_5_	Mo(OR_F6_)_5_	Mo(OR_F9_)_5_
*e*″ (nm)	1596	1523	1763	1799
*e*′ (nm)	662	676	707	873
*e*′ (nm)	599	593	668	736
*g*_*x*_	1.960	1.956	1.952	1.946
*g*_*y*_	1.930	1.932	1.922	1.891
*g*_*z*_	1.837	1.844	1.819	1.807

All molybdenum complexes have a distorted TBP geometry due to the Jahn–Teller effect driven by the singly occupied d_*xz*_ orbital. This lifts the degeneracy of the d_*xz*_ and d_*yz*_ set. An important note here is that the principal symmetry axis is no longer formally the *z* axis, but instead aligns with one ligand in the equatorial plane. However, since from a molecular point of view these complexes are best described as distorted TBP, we assign the two axial ligands, *i.e.* the two longest Mo–O bonds, as the molecular *z*-axis. Using the axial ligands of these trigonal bipyramidal (TBP) complexes to define the *z*-axis of the reference frame, we assign the d_*xz*_ orbital as the singly occupied molecular orbital (SOMO), which is aligned with the longest Mo–O_eq_ bond in line with the π-antibonding character of the SOMO. We then analyze the orientation dependence of the *g*-tensor through symmetry-adapted considerations of orbital angular momentum (Fig. 3, see also Fig. S13). This is valid for this Mo(v) system since it contains orbitals localized on a single atom, with spin–orbit coupling (SOC) dominated by a single electron. The *g*-tensor then arises from SOC between the singly occupied molecular orbital and excited virtual orbitals.^33,34^ Perturbation theory provides an expression for the deviation of each principal *g*-value *g*_*i*_ (for *i* = *x*, *y*, *z*) from the free electron value *g*_e_ ≈ 2.0023 as:1
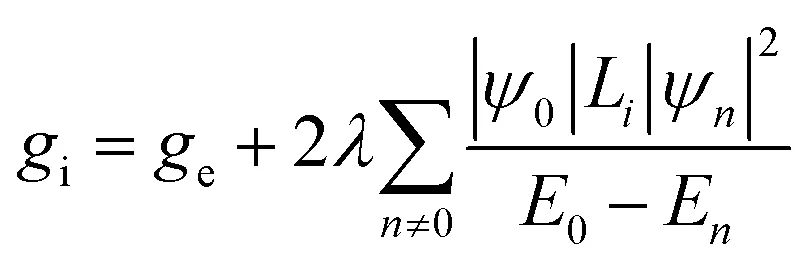
where *ψ*_0_ is the d_*xz*_ SOMO, *ψ*_*n*_ are virtual orbitals, *L*_*i*_ is the angular momentum operator along Cartesian axis *i*, *λ* is the spin–orbit coupling constant and *E*_0_ − *E*_*n*_ is the orbital energy gap.

This expression shows that the *g*-tensor anisotropy arises from coupling the SOMO with virtual excited states through spin–orbit coupling, by orbital angular momentum contributions scaled by their energy separation. Orbitals that are close in energy and symmetry-allowed to mix with the SOMO contribute most strongly, whereas energetically remote or symmetry-forbidden states contribute little. Consequently, every principal *g*-tensor component reflects how strongly the SOMO interacts, *via* the angular momentum operator, with different sets of virtual orbitals along three axes. For atomic-like d-orbitals, the relevant angular momentum coupling coefficients are well established, as only specific pairs of d orbitals mix under *L*_*x*_, *L*_*y*_ or *L*_*z*_. These relationships are quantified in the magic pentagon (Scheme S1) and allow us to identify which d orbitals couple to the SOMO along each axis.^35^

In this distorted TBP geometry, rotation around the *z*-axis transforms the d_*xz*_ SOMO into the d_*yz*_ orbital, which is the Lowest Unoccupied Molecular Orbital (LUMO), *via* the angular momentum operator *L*_*z*_ (Fig. 3a). Therefore, the *g*_*z*_ component is sensitive to the energy gap between d_*xz*_ and d_*yz*_ and reflects the SOMO–LUMO separation. Rotation around the *x*-axis couples d_*xz*_ to a nearly degenerate pair of d_*xy*_ and d_*x*^2^−*y*^2^_ orbitals, which lie at intermediate energy in the ligand field splitting. Hence, *g*_*x*_ reflects the magnitude of interaction with these orbitals. Finally, rotation around the *y*-axis couples d_*xz*_ with d_*z*^2^_—the highest-lying d orbital in the TBP splitting pattern—making *g*_*y*_ sensitive to the energy difference between d_*xz*_ and d_*z*^2^_, and thereby a probe of the axial ligand field strength. Through eqn (1), this can also be expressed as *g*_*y*_ = *g*_e_ − 6*λ*/(*E*(d_*xz*_) − *E*(d_*z*^2^_)), with an estimated spin–orbit coupling constant of 0.1 eV, and an energy gap of 6 eV (Fig. 3c), this matches the observed *g*_*y*_ values of around 1.9, supporting the use of qualitative orbital analysis to link the EPR *g* tensor to the electronics of the complexes.^36^ Visualizing the calculated *g*-tensor confirms that in all complexes, *g*_*z*_ is the smallest component, lying along the axial ligands, *g*_*y*_ is intermediate, and *g*_*x*_ is the largest (Fig. 3b). The smallest shift in *g*_*y*_ for the F_3_-substituted complex (closest to the free electron value) indicates that the d_*xz*_–d_*z*^2^_ energy gap is largest for this system. Because Δ*g*_*y*_ ∝ 1/(*E*_d*z*^2^_ − *E*_d*xz*_), a smaller deviation of *g*_*y*_ from *g*_e_ implies a larger orbital splitting, *i.e.*, a stronger axial ligand field.

The experimentally observed *g*_*y*_ values follow the same trend: *g*_*y*_(F_3_) > *g*_*y*_(F_0_) > *g*_*y*_(F_6_). This suggests that partial fluorination enhances the axial ligand field beyond both the non-fluorinated and fully fluorinated extremes.

The stronger axial field in the F_3_ complex leads to increased orbital splitting and reduced spin–orbit coupling contributions to *g*_*y*_, consistent with a more covalent, directional bonding interaction. DFT-calculated energies of the ligand field window and their associated d-orbitals (Fig. 3c) support this orbital assignment, confirming that the observed *g*-tensor anisotropy can be rationalized in terms of the ligand field-driven d-orbital splitting pattern. This provides experimental evidence that the homoleptic Mo(OR_F3_)_5_ complex has the largest ligand field splitting, and fluorination of *tert*-butoxide counterintuitively causes it to become a stronger field ligand.

### Electronic origin of the non-monotonic fluorination effect

**Fig. 4:**
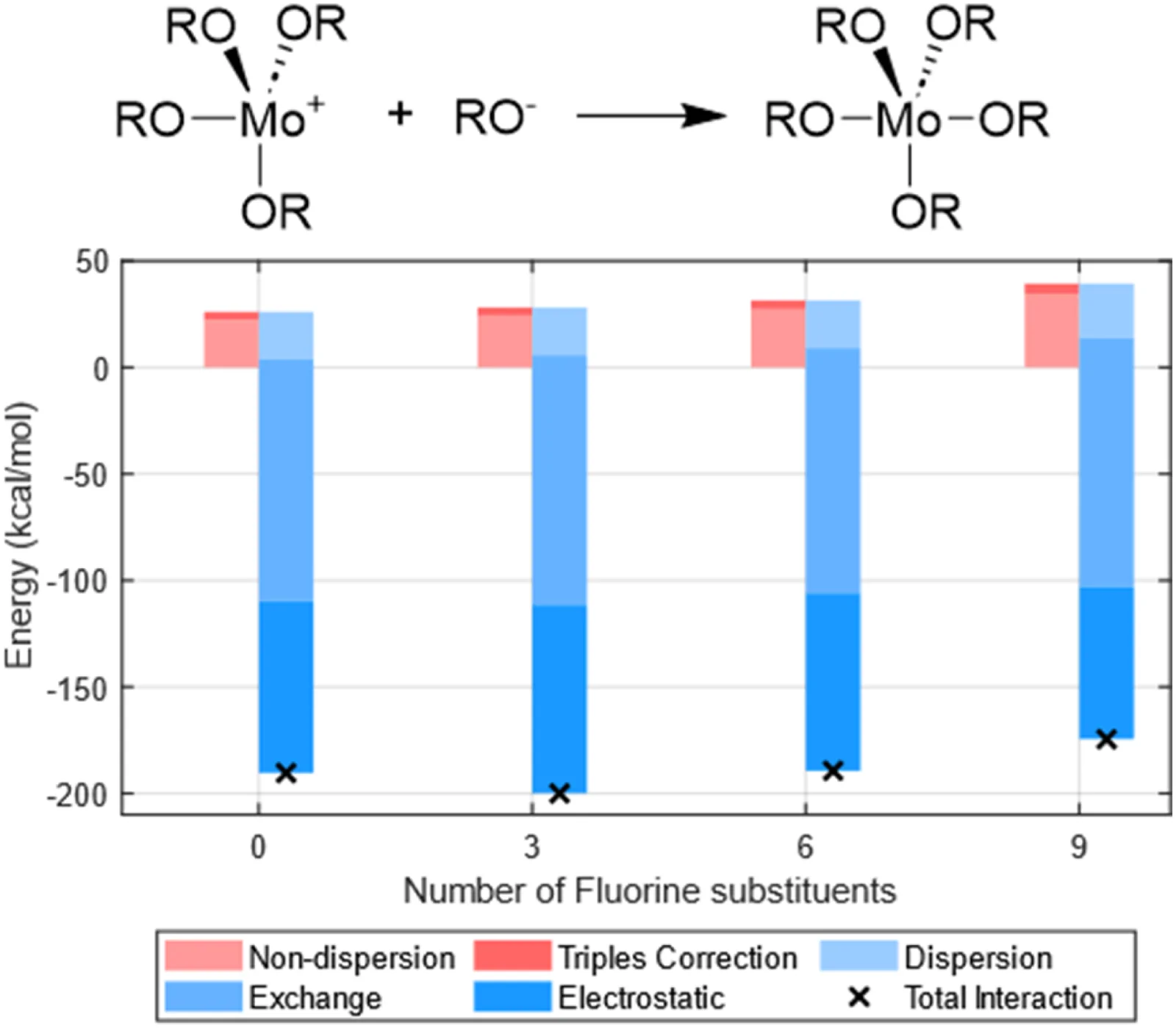
Local energy decomposition of the ligation of a butoxide ligand to a Mo(O^*t*^Bu-F_*n*_)_4_^+^ fragment, calculated at the DLPNO-CCSD(T)/def2-TZVP level of theory. Total contributions (crosses) are broken down into electrostatics, exchange, dispersion, correlation and correction terms.

In order to further evaluate whether partial fluorination causes the metal–ligand bond to become stronger, we next perform fragment-level Local Energy Decomposition (LED) for axial ligand coordination to the complex at the DLPNO-CCSD(T) level of theory (Fig. 4). The axial ligand was chosen as a consistent site across the series, with the primary aim of determining whether fluorination introduces significant dispersion interactions in the outer ligand sphere. The total interaction energy is decomposed into electrostatic, exchange, non-dispersion, dispersion, and correlation terms.^37^ The electrostatic component has an energetic penalty for distorting and polarizing the orbitals (Δ*E*_elprep_) and a gain from coulombic attraction (*E*_elstat_). Initial fluorination strengthens electrostatic interactions significantly, becoming more negative by about 40 kcal mol^−1^ from O^*t*^Bu to OR_F3_, accompanied by an increased electronic preparation cost (Δ*E*_elprep_ − *E*_elstat_ = −88 kcal mol^−1^, *versus* −80 kcal mol^−1^ for F_0_). However, further fluorination to OR_F6_ shows a moderate reversal in this trend (−84 kcal mol^−1^), with a further decrease upon perfluorination to OR_F9_ (−72 kcal mol^−1^). Exchange and dispersion energies remain relatively stable with increasing fluorination, indicating that electrostatic terms drive the observed differences in total interaction energy.

Initially, we inspect trends in common charge descriptors from DFT (B3LYP/def2-TZVPD/d3) population analyses on optimized alkoxide ligands, using Boltzmann-averaged properties from GOAT-generated conformers. The Hirshfeld charge on oxygen becomes less negative from −0.575 (O^*t*^Bu) to −0.512 (OR_F9_), indicating reduced electron density with fluorination.

NBO analysis shows corresponding decreases in lone pair occupancy (1.851 → 1.837 → 1.823 → 1.809*e*) and orbital energy, consistent with electron withdrawal. Likewise, the isotropic polarizability drops linearly from 88 to 70 a.u., reflecting a stiffer electron cloud. Overall, these descriptors point to a linear decrease in σ-donor strength as fluorination increases, which would not explain the EPR and LED results.

**Fig. 5:**
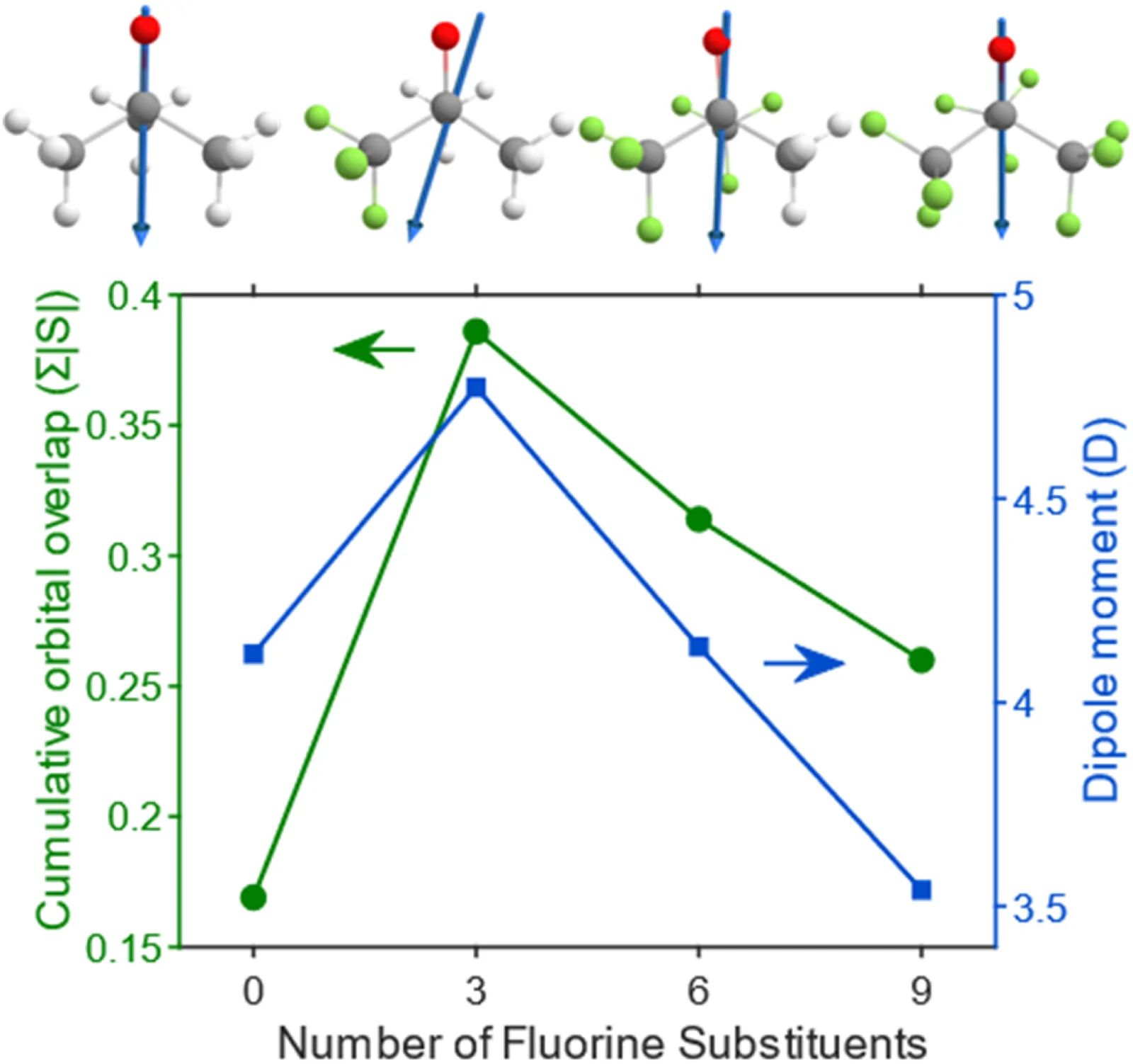
Cumulative orbital overlap of canonical donor/acceptor orbitals (left) of the axial ligand and MoX_4_^+^ fragment. Boltzmann-weighted gas-phase dipole moments for F_*n*_-*tert*-butoxide ligands, calculated at the B3LYP/def2-TZVPD level (right).

We next turn our analysis to the shape and overlap of orbitals. To start, we directly quantify the orbital interaction quality by computing cumulative donor–acceptor overlap integrals between the two highest occupied p-type lone pair orbitals of the alkoxide fragment (HOMO and HOMO−1) and the lowest unoccupied σ- and π-type acceptor orbitals (LUMO and LUMO+1) on the putative Mo(OR)_4_^+^ fragment using canonical orbitals (B3LYP/def2-TZVPD). As this analysis is based on orbitals of isolated, formal fragments, it is intended only as a comparative descriptor of overlap. Summing over the four most relevant pairs yields a clear maximum overlap at F_3_, nearly double that for the parent *tert*-butoxide (Fig. 5, green). Further fluorination weakens this effect, implying that orbital shape is governing this overlap. However, as the orbital overlap is not metal-independent, we sought descriptors sensitive to the orbital asymmetry around the alkoxide ligands in the absence of a metal. The gas-phase dipole moment of the free ligand itself reaches its highest value at F_3_ (4.8 D), larger than both the non-fluorinated (4.1 D) and fully fluorinated (3.5 D) cases (Fig. 5, blue). The nonlinear trend in dipole moment indicates that the nonlinear trends found in the complexes are intrinsic properties of the ligand. We extended this analysis *in silico* to all 20 possible fluorotertbutoxide ligands, exploring the positional effects of fluorination on donor properties (see the SI). However, as dipole moment is a system descriptor and difficult to link to individual orbital contributions, we therefore sought a local descriptor of symmetry around oxygen by computing the ^17^O chemical shielding tensor.

**Fig. 6:**
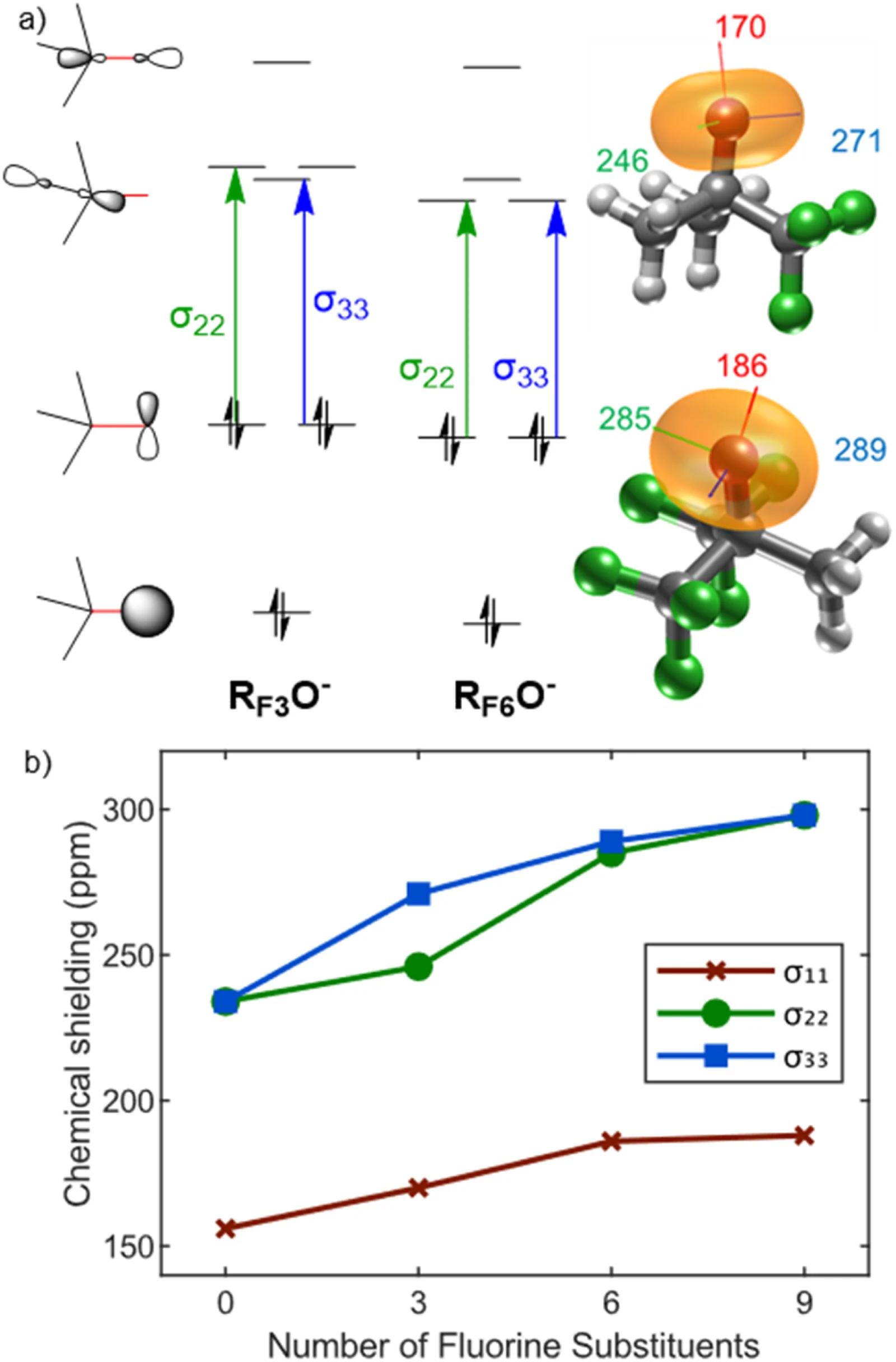
(a) NBO-computed orbital energies and visualized shielding tensors for R_F3_O^−^ and R_F6_O^−^. (b) Computed principal components of the shielding tensors of fluorinated *tert*-butyl alkoxides, computed at the B3LYP/x2c-TZVP level of theory.

The NBO analysis paints a clear picture of the valence orbitals in all *tert*-butoxides. The filled orbitals consist of a low-lying s-type lone pair and two degenerate valence lone pairs. The empty orbitals consist of a set of three C–C antibonding orbitals and a higher lying C–O antibonding orbital. The three C–C antibonding orbitals are degenerate in the case of ^*t*^BuO^−^ and R_F9_O^−^, and nondegenerate for R_F3_O^−^ and R_F6_O^−^ (Fig. 6). We then move on to compute the ^17^O NMR signature as this is highly sensitive to the symmetry and electronic structure around the oxygen atoms.^38–40^ Visualizing the computed ^17^O shielding tensor reveals the *σ*_11_ component to be parallel to the C–O bond and tilted slightly off-axis for R_F3_O^−^ and R_F6_O^−^ (Fig. 6, red). The principal components of the computed ^17^O shielding tensors reveal that the axial symmetry of *tert*-butoxide and perfluoro *tert*-butoxide is lifted for R_F3_O^−^ and R_F6_O^−^. This loss of symmetry is evident from the differentiation of *σ*_22_ and *σ*_33_ (Fig. 6).

Visualizing these components shows that they are roughly perpendicular to the C–O bond and that the *σ*_22_ component is dominant in this anisotropy, aligning with one valence lone pair.

To probe the underlying orbital interactions, we also examine the NBO orbital energies and the energy gaps between the oxygen valence lone pairs (LP2 and LP3) and the relevant C–C σ* orbitals. These LP → σ* energy gaps correlate linearly with the *σ*_22_ and *σ*_33_ components in the fluorination series. In R_F3_O^−^, the C–C σ* corresponding to the CF_3_ moiety is slightly lower in energy and selectively mixes with one of the valence lone pairs, reflected in the marked increase in the *σ*_33_ component.

In R_F6_O^−^, both valence lone pairs have a C–CF_3_ σ* orbital available for mixing, reflected in the marked increase in the *σ*_22_ component. Thus, the presence of distinct low-energy C–CF_3_ σ* acceptor orbitals provides directional channels through which electron withdrawal is transmitted. This can be understood as a hyperconjugation effect. While negative hyperconjugation (LP → σ*) is established as a driver for ligand-field strength in silanolates and fluorinated alkoxides through reducing competitive donation to the metal center, these effects are typically treated without a specific orientation.^41–43^ However, our NBO and NMR analyses demonstrate that this effect is directional: in partially fluorinated derivatives such as R_F3_O^−^, the selective mixing of a single valence lone pair creates an electronic asymmetry that is not captured by simple inductive models. As a result, the oxygen lone pairs respond anisotropically towards the available antibonding orbitals.

This information now makes clear why, in Mo(OR_F3_)_5_, the CF_3_ substituent adopts an antiperiplanar configuration to molybdenum in the crystal structure (Fig. 2). As a result, the oxygen lone pairs respond anisotropically towards the available antibonding orbitals. This limits the electron withdrawing effect to an area that is not available for π-bonding, leaving the relevant valence orbitals for the Mo–O bond untouched.

### Conformational effects in a Schrock-type olefin metathesis catalyst

**Fig. 7:**
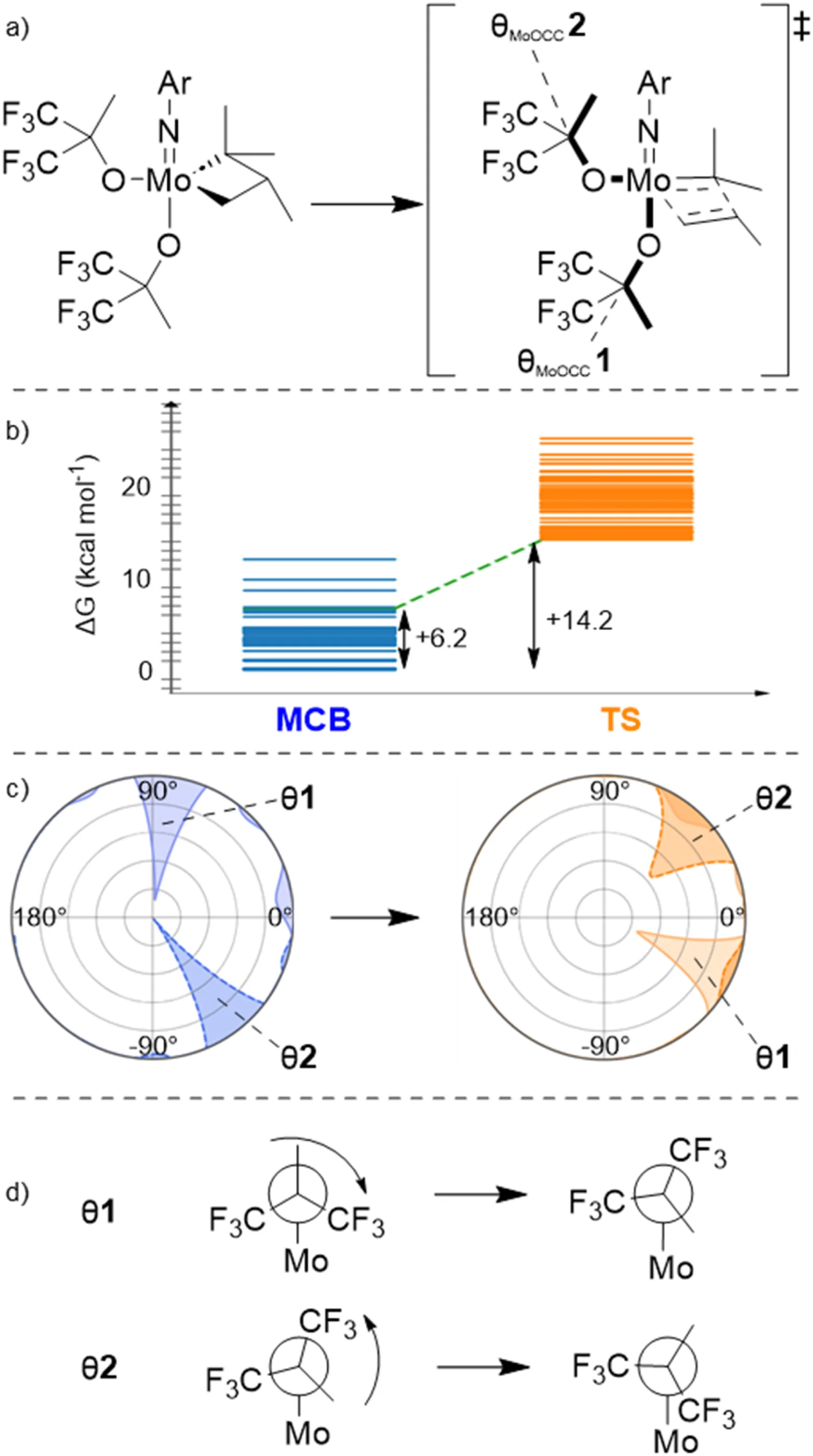
Conformational analysis of the ground state and transition state in the Schrock olefin metathesis catalyst. (a) MCB ground state and transition state; dihedral angles investigated are shown in bold. (b) Energy diagram of all conformers found for ground and transition states. (c) Dihedral plots showing Boltzmann distribution of Mo–O–C–CH_3_ angles in the ground state and transition state. (d) Ground to transition state rotations of alkoxides.

Having established that the π-bonding of partially fluorinated alkoxides is dependent on the alignment, we were interested to know how this could influence relevant catalytic intermediates of olefin metathesis. Therefore, we carried out an extensive conformer analysis of both the metallacyclobutane ground state and the transition state for cycloreversion, one of the key steps in olefin metathesis, to investigate whether the alkoxides prefer a particular alignment and how this conformational preference could affect the ease of cycloreversion at specific points on the potential energy surface. In order to investigate this, a GOAT conformer search was performed on the reaction of a Schrock catalyst with an olefin (Fig. 7a, full details in the SI). This provided 61 conformers for the ground state and 86 for the transition state (Fig. 7b). These conformers were then further optimized at the r2scan0 level of theory to gain accurate energies, which were used to provide Boltzmann populations of the conformers.

**Fig. 8:**
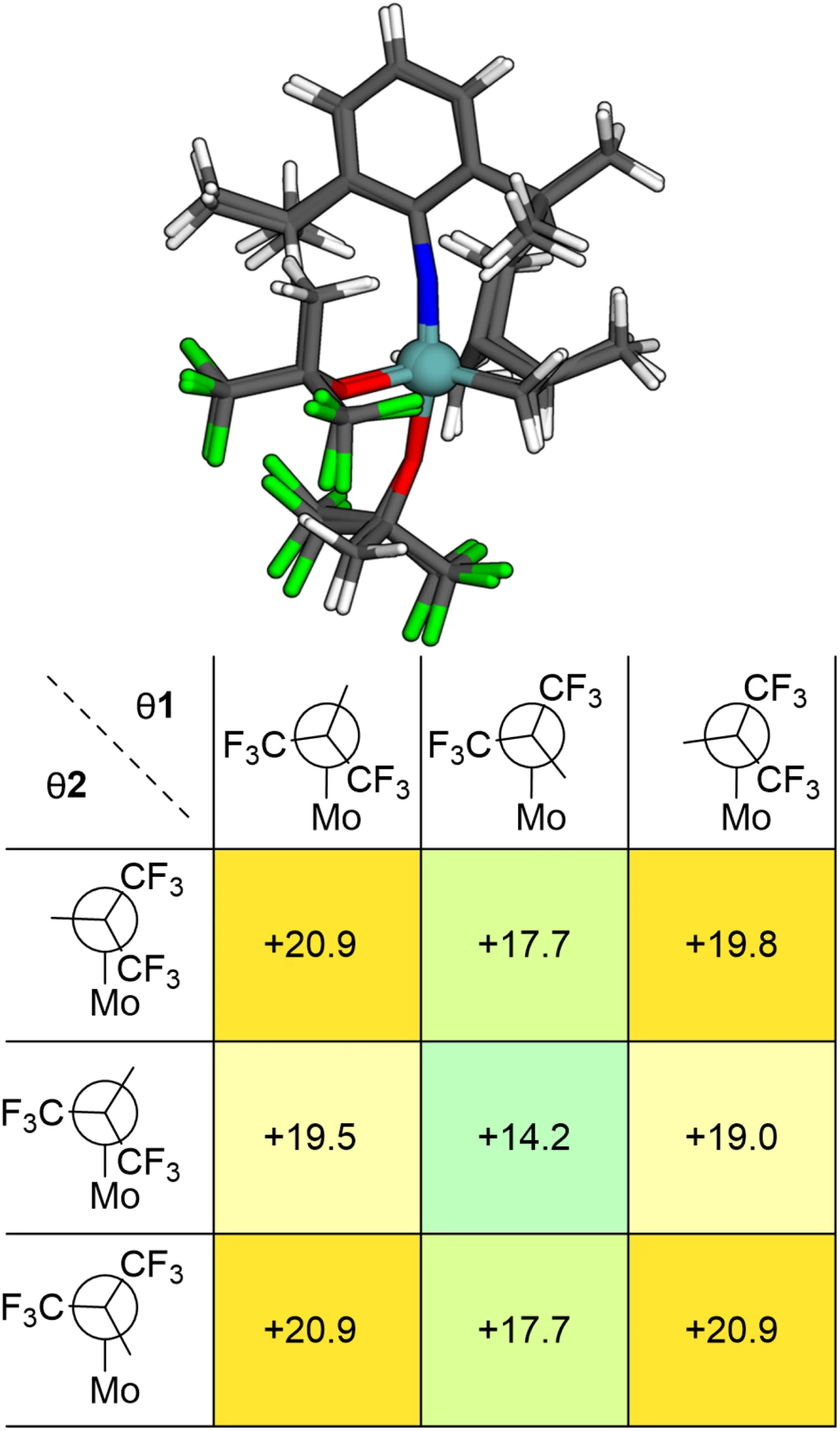
(a) Overlay of the ground state conformer of the molybdenum imido metallacyclobutane (+6.2 kcal mol^−1^) closest to the lowest transition state geometry (+15.6 kcal mol^−1^). (b) Transition state energies as a function of the dihedral angle *θ*1 and *θ*2.

The dihedral Mo–O–C–CH_3_ angles *θ*1 (alkoxide *trans* to imido, Fig. 7a) and *θ*2 (alkoxide *trans* to MCB, Fig. 7a) were then evaluated for each conformer, and, using the Boltzmann populations, this provided the favoured orientations for the ground and transition states. These are displayed in Fig. 7c in radial plots, where the closer to the center an angle is, the more populated that conformer is. For the MCB ground state, minima are observed at 90° for *θ*1 and −60° for *θ*2. Conversely, the lowest transition state lies +14.2 kcal mol^−1^ above the lowest ground state where these alkoxides are rotated by 90° and 120°, respectively, while the ground state conformer with a geometry closest to the transition state (RMSD = 1.08 Å) lies +6.2 kcal mol^−1^ higher in energy with the alkoxides rotated to other specific positions (Fig. 8a). This higher energy intermediate is shown overlaid with the lowest conformer of the transition state, which makes it clear that from this intermediate geometry, only the bond-breaking event needs to occur for turnover.

Then, we used the generated conformer ensemble to search for the transition states with just one or both of the alkoxides rotated by approximately 120° in order to investigate the effect of alignment of the R_F6_O^−^ group on the potential energy surface. Starting from the lowest transition state geometry, with an energy of +14.2 kcal mol^−1^, clockwise or counterclockwise rotation of dihedral *θ*1 *trans* to the imido by 120° leads to an increase of approximately 5 kcal mol^−1^ in the Gibbs free energy to +19.0 or +19.5 kcal mol^−1^, respectively (Fig. 8b). Clockwise or counterclockwise rotation of dihedral *θ*2 *trans* to the metallacyclobutane by 120° leads to an increase of approximately 3 kcal mol^−1^ to +17.7 kcal mol^−1^ (extended discussion in the SI). Rotation of both alkoxides increases the energy by +6.4 kcal mol^−1^ on average.

This energy difference is remarkably close to the +6.2 kcal mol^−1^ difference in energy between the lowest ground state and the ground state most closely resembling the transition state (Fig. 8a). With negligible differences in sterics of CH_3_ and CF_3_ groups,^44^ we can only assign this to electronics, providing support for the directionality of electron withdrawing by partially fluorinated *tert*-butyl groups. The combined effect of alignment of the two alkoxides in the Schrock catalyst is able to account for differences in the transition state barrier of 6 kcal mol^−1^, likely pointing to the need for conformation changes for efficient catalysis.

## Conclusions

Overall, our combined spectroscopic and computational analysis reveals that partial fluorination, specifically the introduction of a single CF_3_ group, enhances the ligand field strength in Mo(OR)_5_ complexes by optimizing the balance between covalent orbital overlap and electrostatic polarization. While global descriptors such as Hirshfeld charge and polarizability suggest a monotonic decrease in donor strength with increasing fluorination, local bonding analyses explain the experimentally observed non-linear behavior driven by electronic reorganization in the ligand. These findings demonstrate that both the number and position of fluorine substituents significantly influence metal–ligand bonding, primarily by altering the spatial localization of the donor orbitals. This is reflected in the Schrock catalyst, which shows a clear preference for specific alkoxide orientations for the metallacyclobutane and in the cycloreversion transition state, requiring conformational changes for an optimal energy landscape. Extensive conformer analysis of the Schrock catalyst reveals the importance of studying dynamic electronic effects in ligands with asymmetric substitution patterns. Given the significance of alkoxide ligands across metathesis, cross-couplings, depolymerization reactivity, and more key areas of reactivity, this is a key insight for designing advanced catalytic systems. This work underscores the importance of evaluating both global and local electronic descriptors to rationalize and predict ligand effects, providing a framework for the rational design of tunable ligands in catalysis and coordination chemistry.

## Author contributions

Felix J. de Zwart: conceptualization, methodology, investigation, formal analysis, data curation, visualization, supervision, project administration, funding acquisition, writing (original draft, review & editing). Noah Gehr: methodology, investigation, writing (review & editing). Sophie W. Anferov: investigation, formal analysis, writing (review & editing). Joël Landis: methodology, investigation, formal analysis, writing (review & editing). Christophe Copéret: supervision, resources, funding acquisition, writing (review & editing).

## Conflicts of interest

There are no conflicts to declare.

## Supplementary Material

SC-OLF-D6SC05403J-s001

## Data Availability

CCDC 2522425 and 2522426 contain the supplementary crystallographic data for this paper.^71*a*,*b*^ A repository dataset has been uploaded at DOI: https://doi.org/10.5281/zenodo.20569296. The authors have cited additional references within the supplementary information (SI).^44–70^ Supplementary information: experimental procedures, characterization of new compounds and computational procedures. See DOI: https://doi.org/10.1039/d6sc05403j.
