# Supplementary material for: Fluorination of alkoxide ligands: neither too much, nor too little for optimal ligand field strength
Source: Chem Sci. 2026 Aug 19;17(40):19247–56. doi: 10.1039/d6sc05403j (PMC13508817; doi:10.1039/d6sc05403j)
Supplement: Supplementary file 1 [file SC-017-D6SC05403J-s001.pdf]

Supplementary Information (SI) for Chemical Science.
This journal is © The Royal Society of Chemistry 2026

Supporting Information for

Fluorination of Alkoxide Ligands: Neither too much, Nor too little for Optimal Ligand Field Strength

Felix J. de Zwart, Noah Gehr, Sophie W. Anferov, Joël Landis, Christophe Copéret

Contents

Supporting Information for ... 1
Contents ... 1
General Procedures ... 2
Synthetic Procedures ... 3
Synthesis of $Mo(O^tBu)_5$ ... 3
Synthesis of $Mo(O^tBu\text{-}F_3)_5$ ... 3
Synthesis of $Mo(O^tBu\text{-}F_6)_5$ ... 3
PXRD Spectra ... 4
NMR Spectra ... 5
EPR Spectra ... 7
Crystallographic Details ... 9
Computational Details ... 11
DFT Population Analyses ... 15
Positional effects on fluorination ... 16
Coordinates of Calculated Structures ... 18
References ... 22

General Procedures

All syntheses were performed using standard Schlenk techniques or in a glovebox in oven-dried glassware under an atmosphere of purified argon. Solvents were obtained from an MBraun solvent purification system and degassed using the freeze-pump-thaw method. Standard chemicals were purchased from ABCR, Acros, Aldrich, Fluka, and Strem. $LiOR_{F3}$, $LiOR_{F6}$ were synthesized according to literature.[1] MoCl4(tht)2 was synthesized according to literature.[2] UV-Vis-NIR spectra were measured on a Cary 5000 spectrometer in dual beam mode with a 1 cm path length cuvette against a reference of pure solvent.

NMR spectra were recorded on a Bruker Avance 300 spectrometer at 298 K. Chemical shifts δ are reported in parts per million (ppm) using residual solvent signal. Multiplicities of the signals are designated by s (singlet), d (doublet), t (triplet), and m (multiplet). Absolute values of coupling constants *J* are given in Hertz (Hz).

EPR spectra were recorded on a magnettech MiniScope MS5000 with a frequency of 9.45 GHz. Pure compounds were dissolved in toluene and frozen in liquid N_2 to form a good glass. The experimental data was fit using EasySpin 6.0.5 in MATLAB R2024b with the CW-EPR plugin.[3,4]

Single crystals were obtained by recrystallization from *n*-pentane at −35 °C for $Mo(OR_{F3})_5$ and $Mo(OR_{F6})_5$. Crystals of each compound were placed on a microscopy slide inside an Ar-filled glovebox and covered with a drop of perfluoropolyalkylether oil. The microscopy slide was removed from the glovebox and a suitable crystal was picked under a microscope and transferred to the diffractometer (nitrogen stream at 100 K) on a Rigaku XtaLAB Synergy S diffractometer with Dualflex HyPix-6000HE detector using Cu-Kα radiation. After data collection, structures were solved by intrinsic phasing (SHELXT) and refined by full-matrix least-squares procedures using SHELXL in the Olex2 program suite.[5-8] C-H hydrogen atoms were generated by geometrical considerations, constrained to idealized geometries, and allowed to ride on their carrier atoms with an isotropic displacement parameter related to the equivalent displacement parameter of their carrier atoms.

For $Mo(OR_{F6})_5$ we note a singular A-level alert, resulting from extraneous electron density near the heavy atoms Mo (not compensated for in the collection). However, the bond lengths and geometrical aspects of the core scaffold are still sufficiently high resolution for interpretation. Deposition numbers 2522425 and 2522426 contain the supplementary crystallographic data for this paper. This data can be obtained free of charge via the joint Cambridge Crystallographic Data Centre (CCDC) and Fachinformationszentrum Karlsruhe Access Structures service.

Synthetic Procedures

Synthesis of $Mo(O^{t}Bu)_5$

In an argon-filled glovebox, an oven-dried 50 mL Schlenk flask, equipped with a Teflon-coated magnetic stir bar, was charged with $Mo(THT)_2Cl_4$ (586 mg, 1.41 mmol) and toluene (15 mL) at –35 °C, to give an orange-brown solution. $LiO^{t}Bu$ (368 mg, 4.54 mmol) was added while stirring. The mixture was allowed to warm to ambient temperature and stirring was continued for 3 h to give a brown suspension. The solvent was removed in vacuo and the residue was extracted with pentane (total of 10 mL) and filtered over celite. A green solution was transferred into a flame-dried, argon-filled 500 mL Schlenk flask equipped with a cold finger and the solvent was removed in vacuo. The crude further purified by sublimation in high-vacuum (40 °C water bath, –193 °C cold finger) to yield the product as a fine green powder (124 mg, 0.269 mmol, 19%). Once sublimed, the product is not stable under high vacuum at room temperature and must be kept cold, and collected from the cold finger as quickly as possible. ^{1}H-NMR (300 MHz, CD_2Cl_2) δ 7.28 (broad) ppm. EPR (X-band, -124 °C, pentane, 9.45 GHz) g_{iso} = 1.885, A^{Mo} iso = 129 MHz.

Synthesis of $Mo(O^{t}Bu\text{-}F_3)_5$

In an argon-filled glovebox, a 50 mL Schlenk flask with a Teflon-coated magnetic stir bar was charged with $Mo(THT)_2Cl_4$ (276 mg, 0.66 mmol) and toluene (10 mL), at –35 °C, to give an orange-brown suspension. LiOtBu-F_3 (355 mg, 2.65 mmol) was added all at once while stirring slowly. The mixture was allowed to warm to ambient temperature and stirring was continued overnight to give a brown suspension. The solvent was removed in vacuo and the residue was extracted with n-pentane (total of 10 mL) and filtered over celite. The product was crystallized at –35 °C as blue crystals (163 mg, 0.23 mmol, 35%).

^{1}H-NMR (300 MHz, C_6D_6) δ 13.20 (broad) ppm. ^{19}F-NMR (282 MHz, C_6D_6) δ -82.53 (broad) ppm. EPR (X-band, -170 °C, toluene glass, 9.43 GHz) g_x = 1.832, g_y = 1.901, g_z = 1.934, A^{Mo}[xyz] = [82 210 75] MHz.

For the purpose of the quantification of side products, the filter plug was washed with DCM, evaporated in vacuo and quantified by NMR against a standard of mesitylene which showed the presence of 0.056 mmol $Mo_2(O^{t}Bu\text{-}F_3)_6$.

Synthesis of $Mo(O^{t}Bu\text{-}F_6)_5$

In the glovebox, a 100 mL oven-dried Schlenk flask was charged with $Mo(THT)_2Cl_4$ (796 mg, 1.91 mmol) and toluene (20 mL), at –35 °C, to give an orange-brown suspension. $LiO^{t}Bu\text{-}F_6$ (1234 mg, 6.56 mmol) was added all at once while stirring slowly. The mixture was allowed to warm to ambient temperature and stirring was continued overnight to give a green-brown suspension. After 16 h, the solvent was removed in vacuo and the residue was extracted with n-pentane (total of 10 mL) and filtered over celite. The solution was stored at –35 °C and the product was collected as deep turquoise crystals in batches (434 mg, 0.43 mmol, 23%).

^{1}H-NMR (300 MHz, C_6D_6) δ 19.23 (broad) ppm. ^{19}F-NMR (282 MHz, C_6D_6) δ -70.22 (broad) ppm. EPR (X-band, -169 °C, toluene glass, 9.44 GHz) g_x = 1.777, g_y = 1.887, g_z = 1.923, A^{Mo}y = 316 MHz.

PXRD Spectra

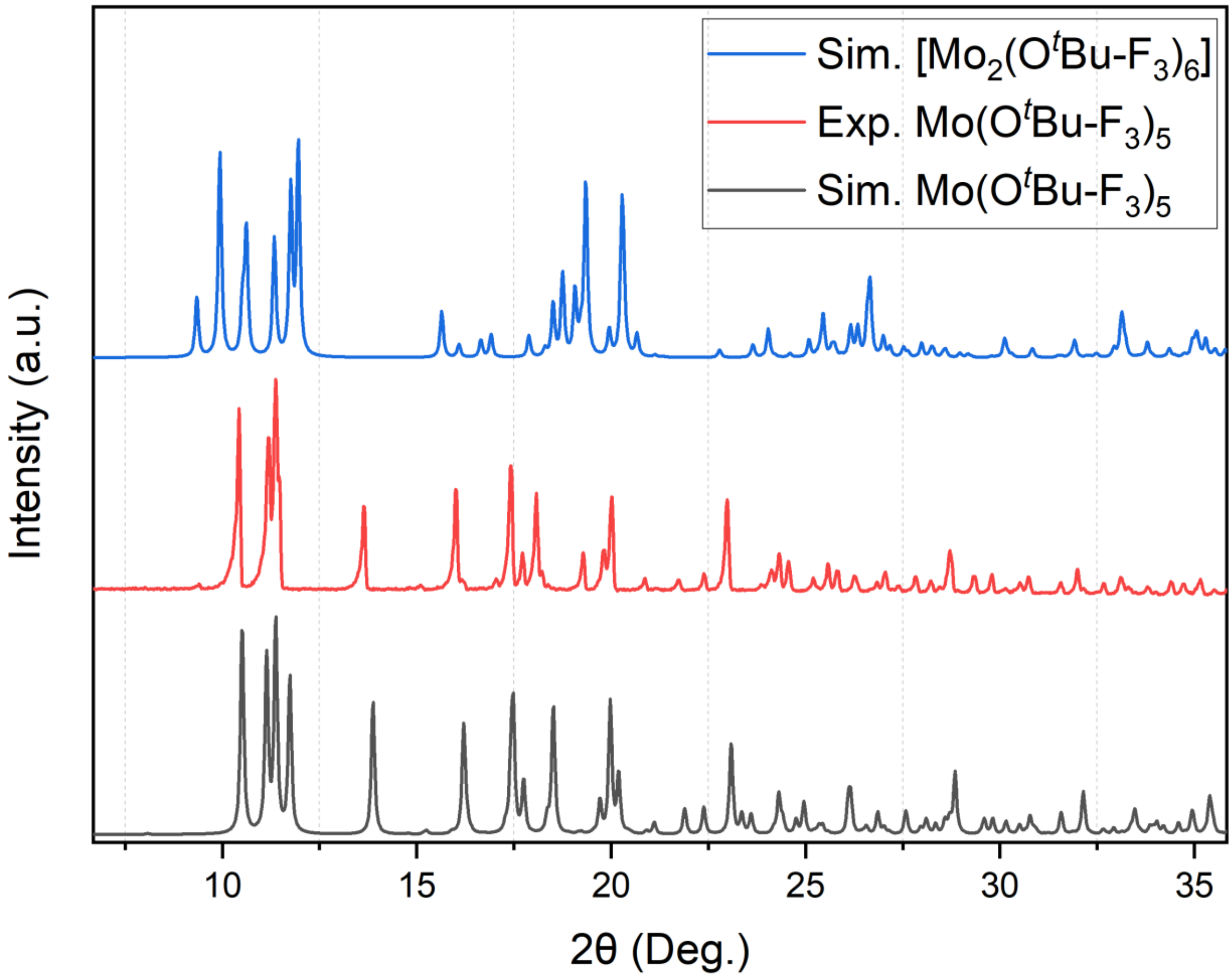

Figure S1. PXRD spectrum for $Mo(O^tBu-F_3)_5$.

NMR Spectra

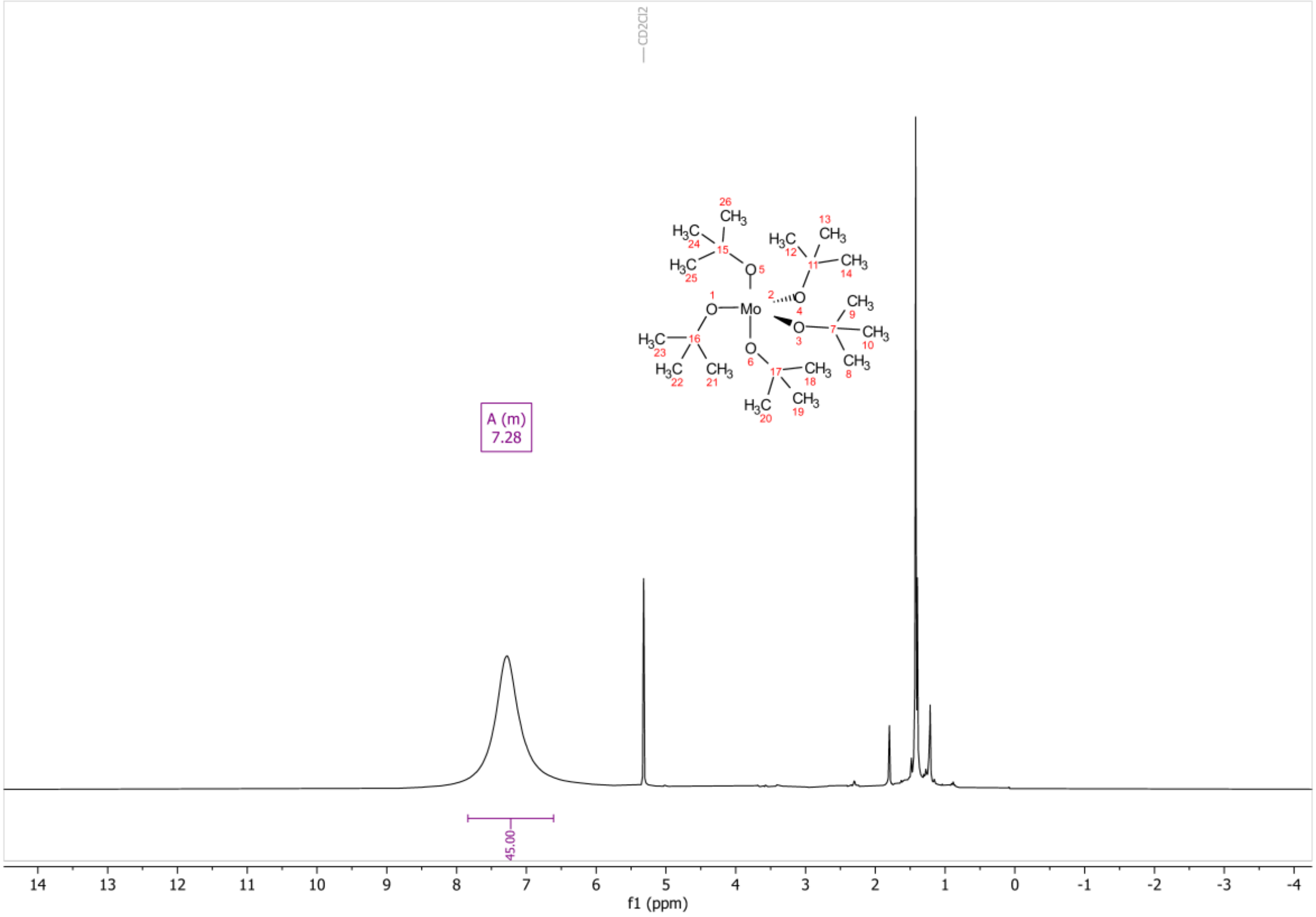

Figure S2. ^{1}H-NMR (300 MHz, CD_2Cl_2) spectrum of $Mo(OtBu)_5$.

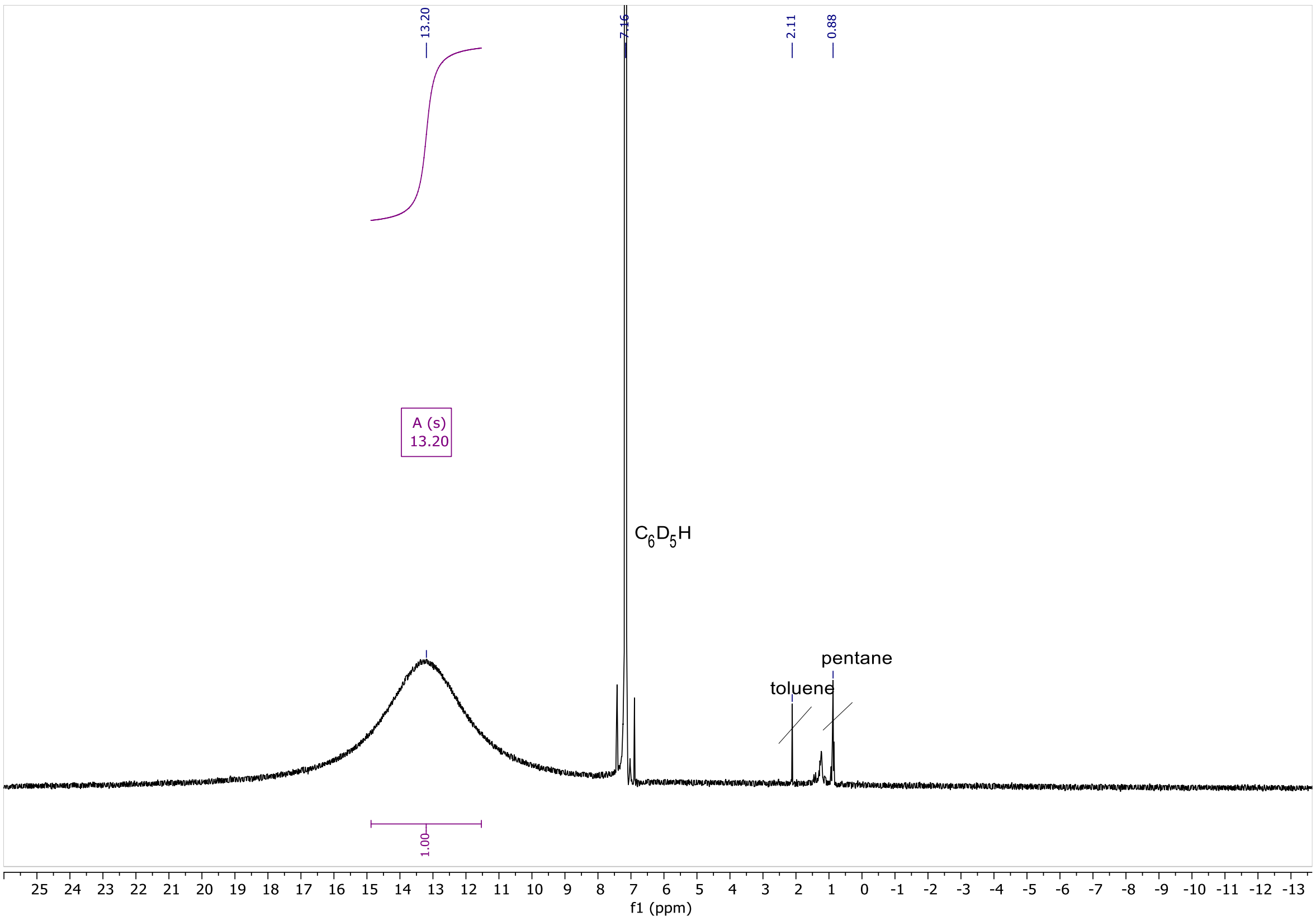

Figure S3. ^{1}H-NMR (300 MHz, C_6D_6) spectrum of $Mo(OtBu\text{-}F_3)_5$.

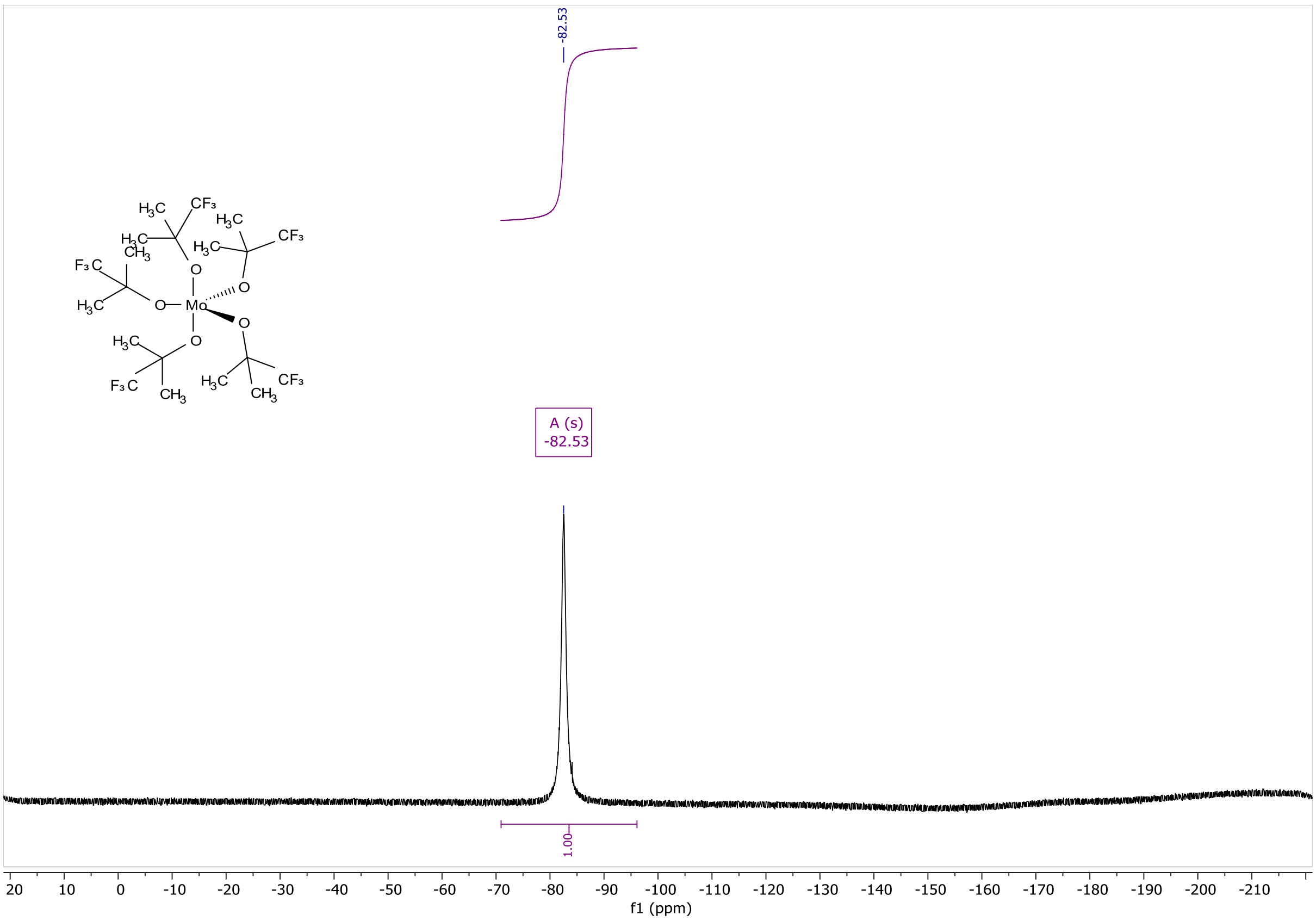

Figure S4. ^{19}F-NMR (282 MHz, C_6D_6) spectrum of $Mo(OtBu\text{-}F_3)_5$.

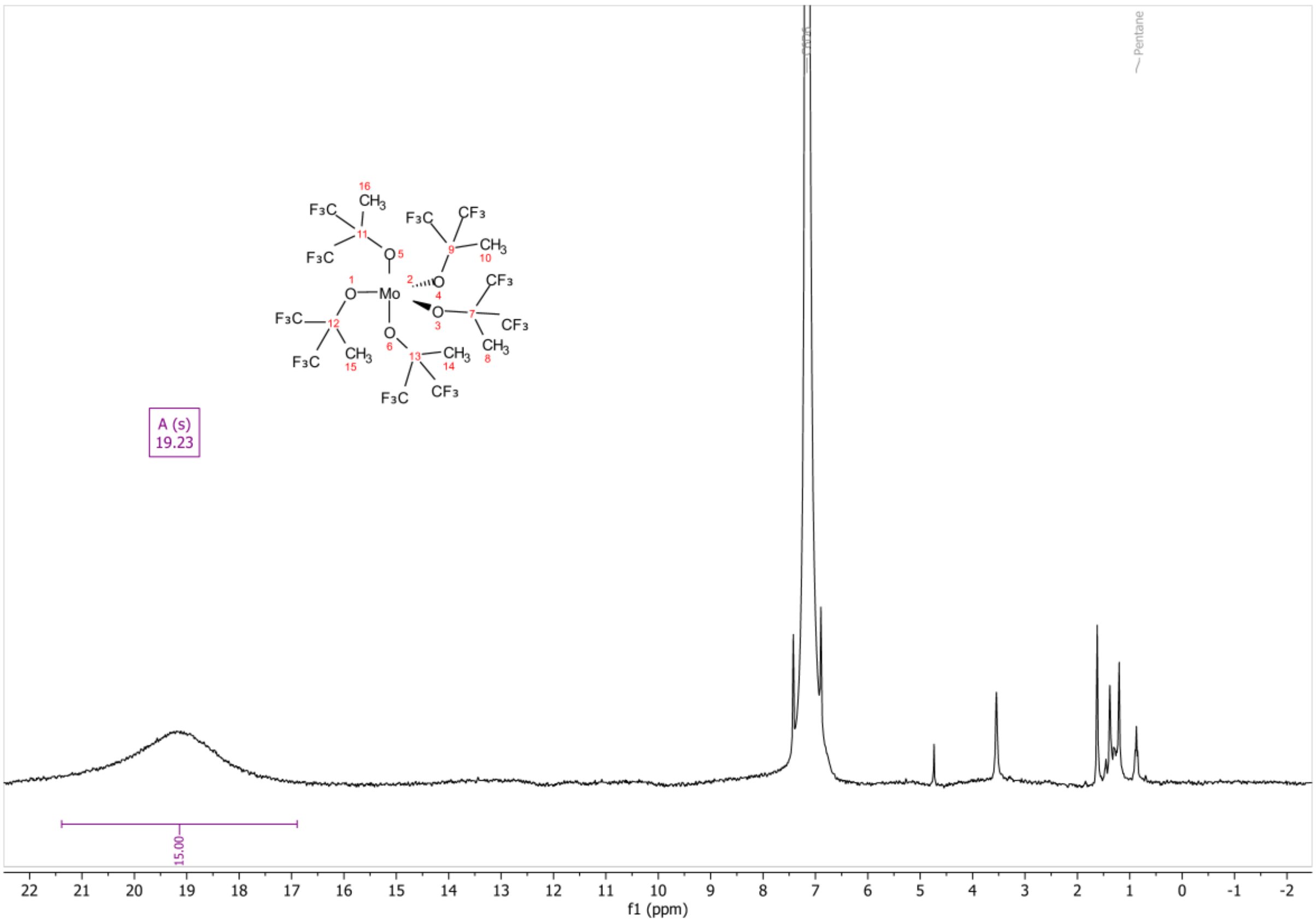

Figure S5. ^{1}H-NMR (300 MHz, C_6D_6) spectrum of $Mo(OtBu\text{-}F_6)_5$.

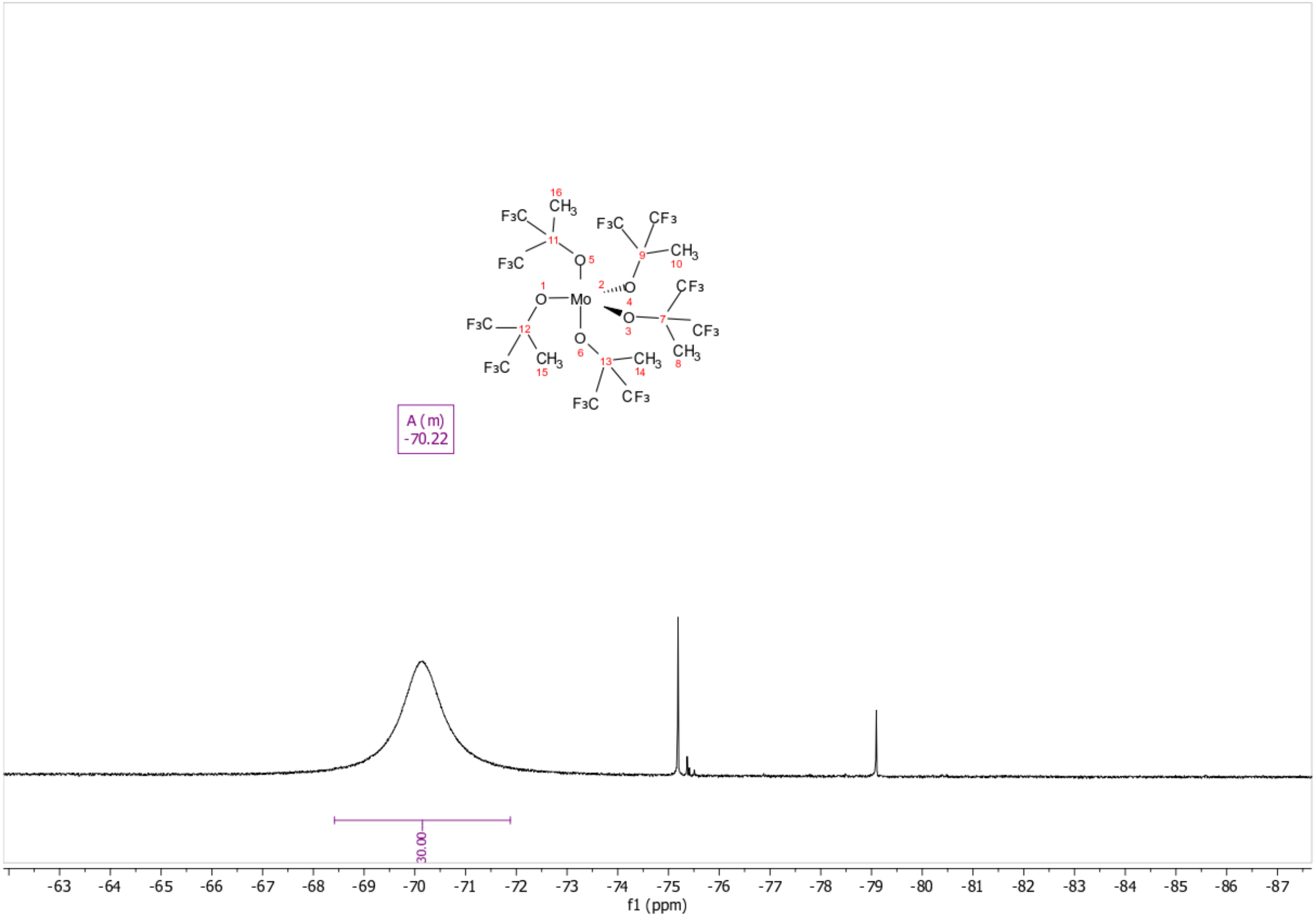

Figure S6. ^{19}F-NMR (282 MHz, C_6D_6) spectrum of Mo(OtBu-F_6)$_5$.

EPR Spectra

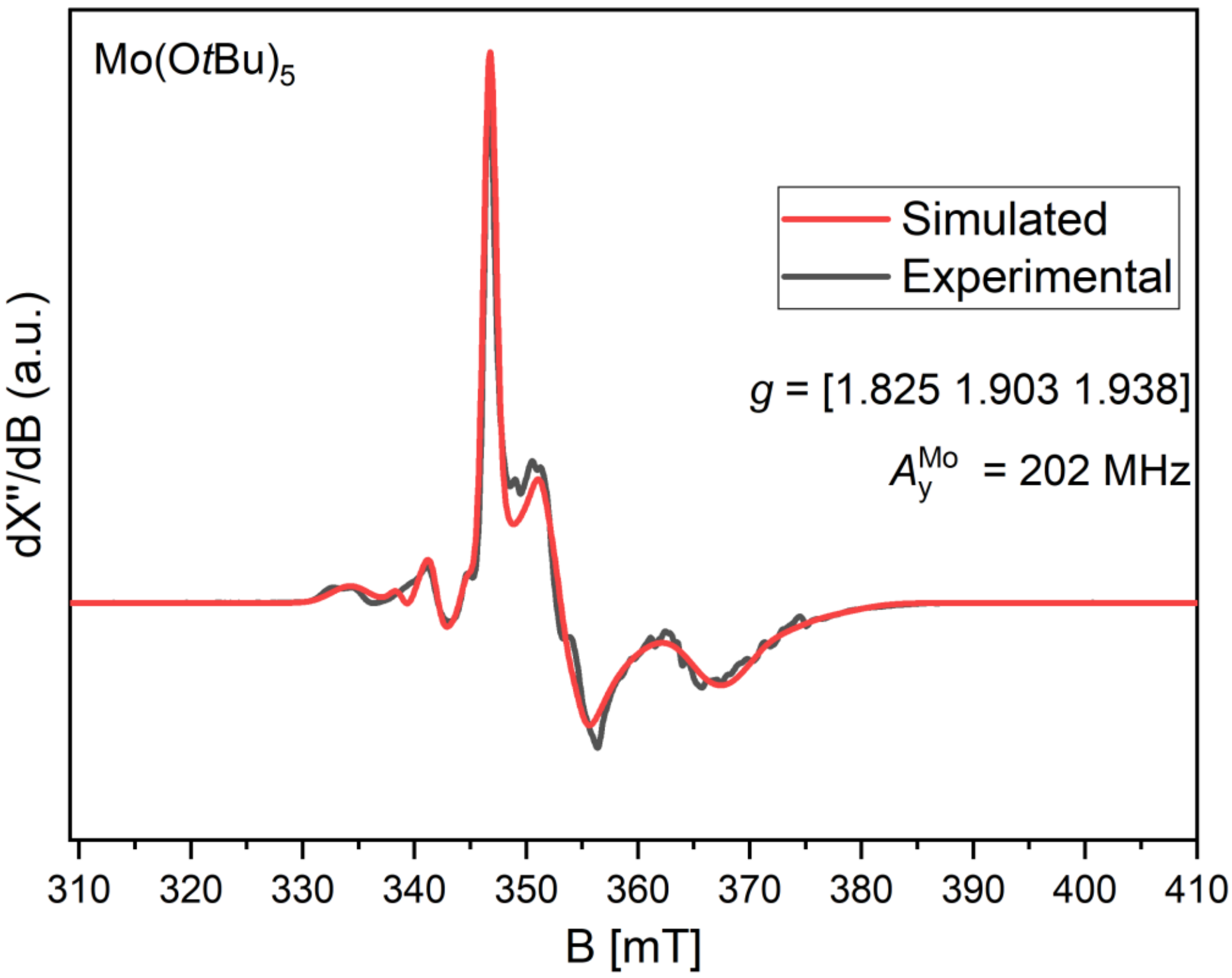

Figure S7. EPR (X-band, -243 °C, toluene, 9.412 GHz) spectrum of Mo(OtBu)$_5$.

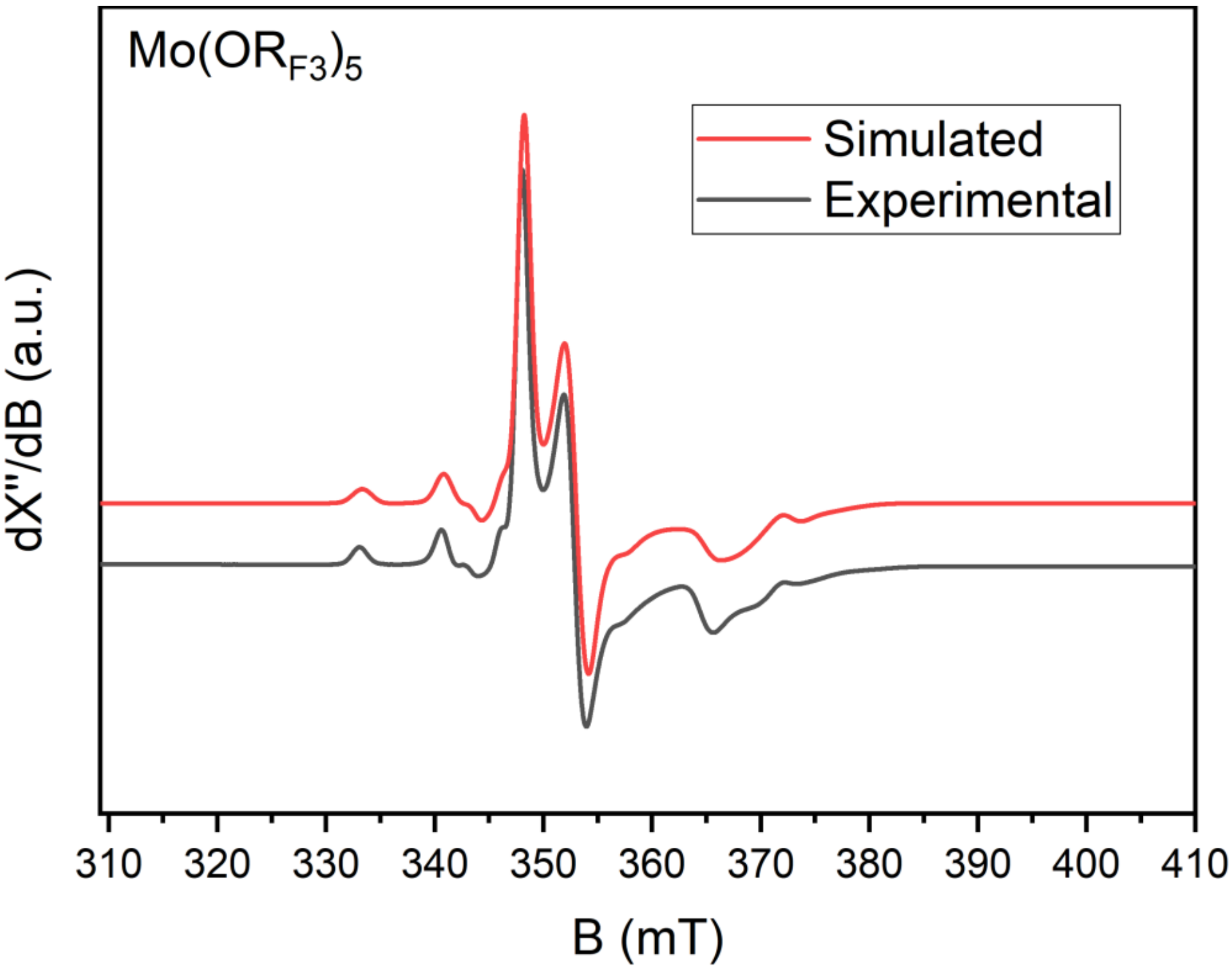

Figure S8. EPR (X-band, -170 °C, toluene, 9.43 GHz) spectrum of Mo(OtBu-F_3)$_5$.

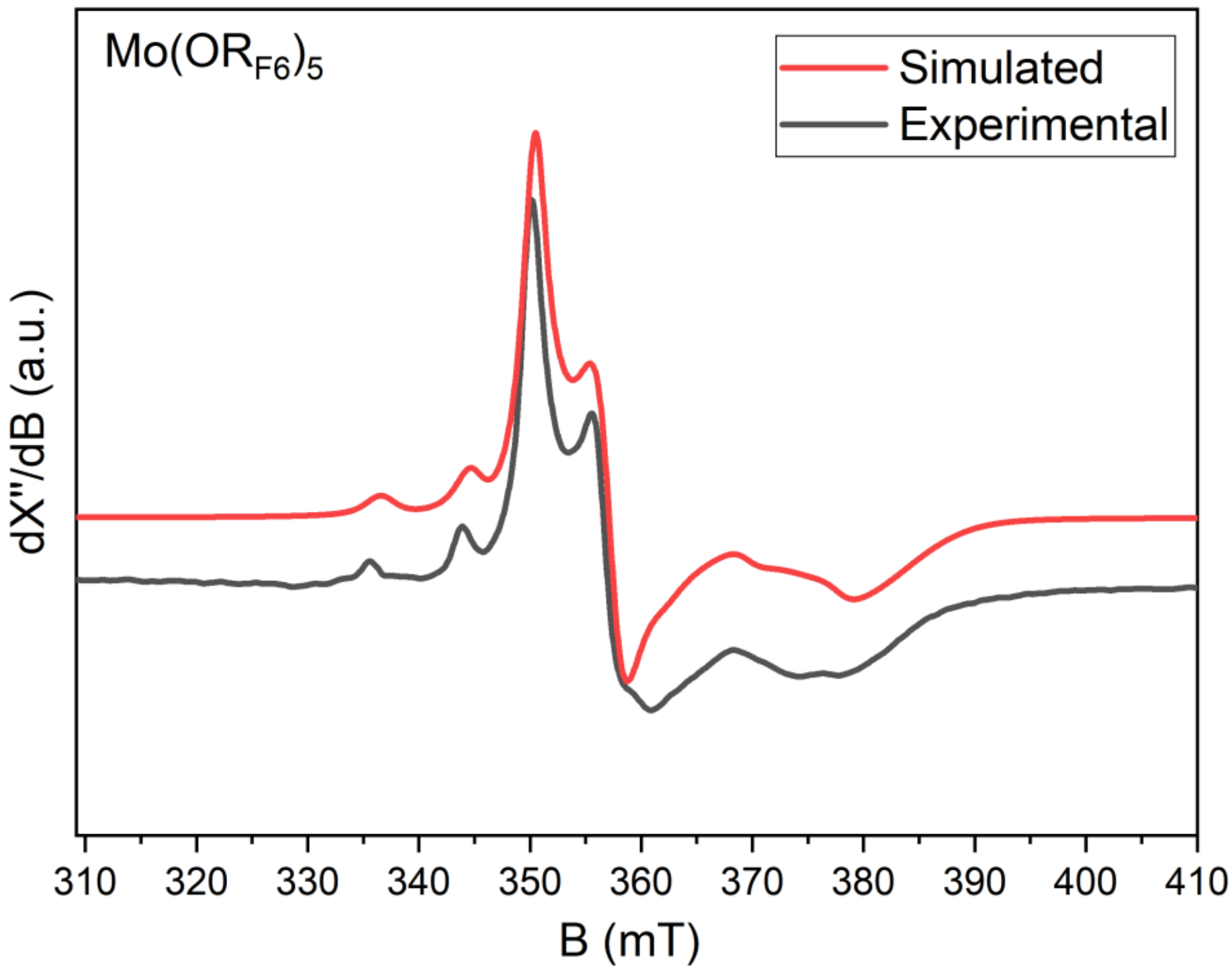

Figure S9. EPR (X-band, -169 °C, toluene, 9.44 GHz) spectrum of Mo(OtBu-F_6)$_5$.

Crystallographic Details

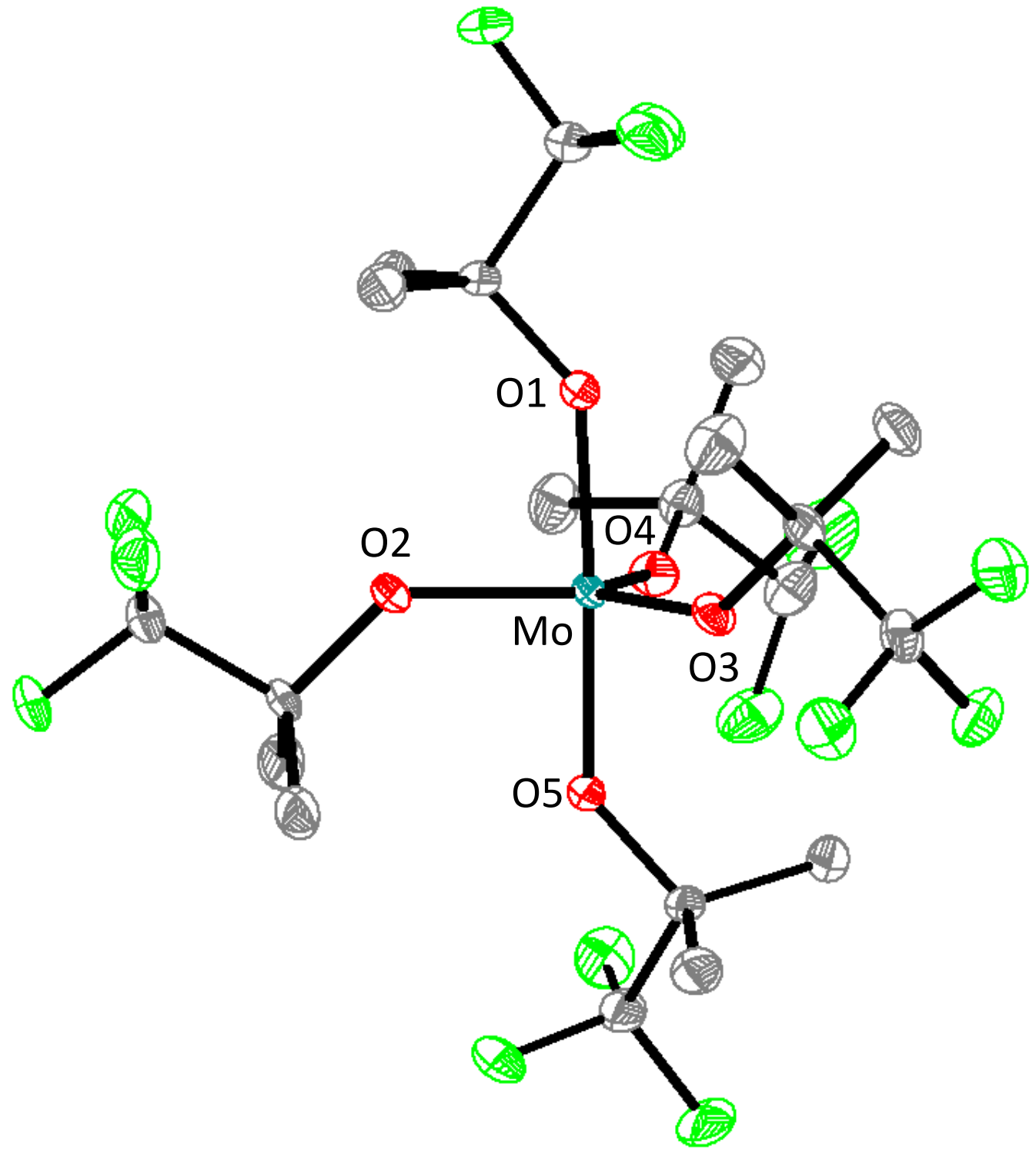

Figure S10. Solid state structure of $Mo(OR_{F3})_5$. Mo (teal), O (red), C (gray), F(green). C-H H-atoms omitted. CCDC 2522426.

Selected bond lengths (Å): Mo-O1: 1.940(1); Mo-O2: 1.881(1); Mo-O3: 1.895(1); Mo-O4: 1.828(2); Mo-O5: 1.927(1); Selected bond angles (°): O1-Mo-O5: 172.91(6); O2-Mo-O3: 120.15(6).

Table S1. SXRD structure of $Mo(OR_{F3})_5$.

Empirical formula	$C_{20}H_{30}F_{15}MoO_5$
Formula weight	731.365
Temperature/K	100.0(2)
Crystal system	monoclinic
Space group	$P2_1/c$
a/Å	15.98048(10)
b/Å	15.06868(8)
c/Å	12.05736(8)
α/°	90
β/°	96.5903(6)
γ/°	90
Volume/Å^3	2884.28(3)
Z	4
ρ_{calc}g/cm^3	1.684
μ/mm^{-1}	4.909
F(000)	1475.4
Crystal size/mm^3	0.983 × 0.489 × 0.373
Radiation	Cu Kα (λ = 1.54184)
2Θ range for data collection/°	5.56 to 160.52
Index ranges	$-20 \le h \le 20$, $-19 \le k \le 19$, $-13 \le l \le 15$

Reflections collected	116381
Independent reflections	6264 [R_{int} = 0.0672, R_{sigma} = 0.0182]
Data/restraints/parameters	6264/0/381
Goodness-of-fit on F^2	1.041
Final R indexes [I>=2σ (I)]	R_1 = 0.0327, wR_2 = 0.0957
Final R indexes [all data]	R_1 = 0.0336, wR_2 = 0.0968
Largest diff. peak/hole / e $Å^{-3}$	0.74/-1.46

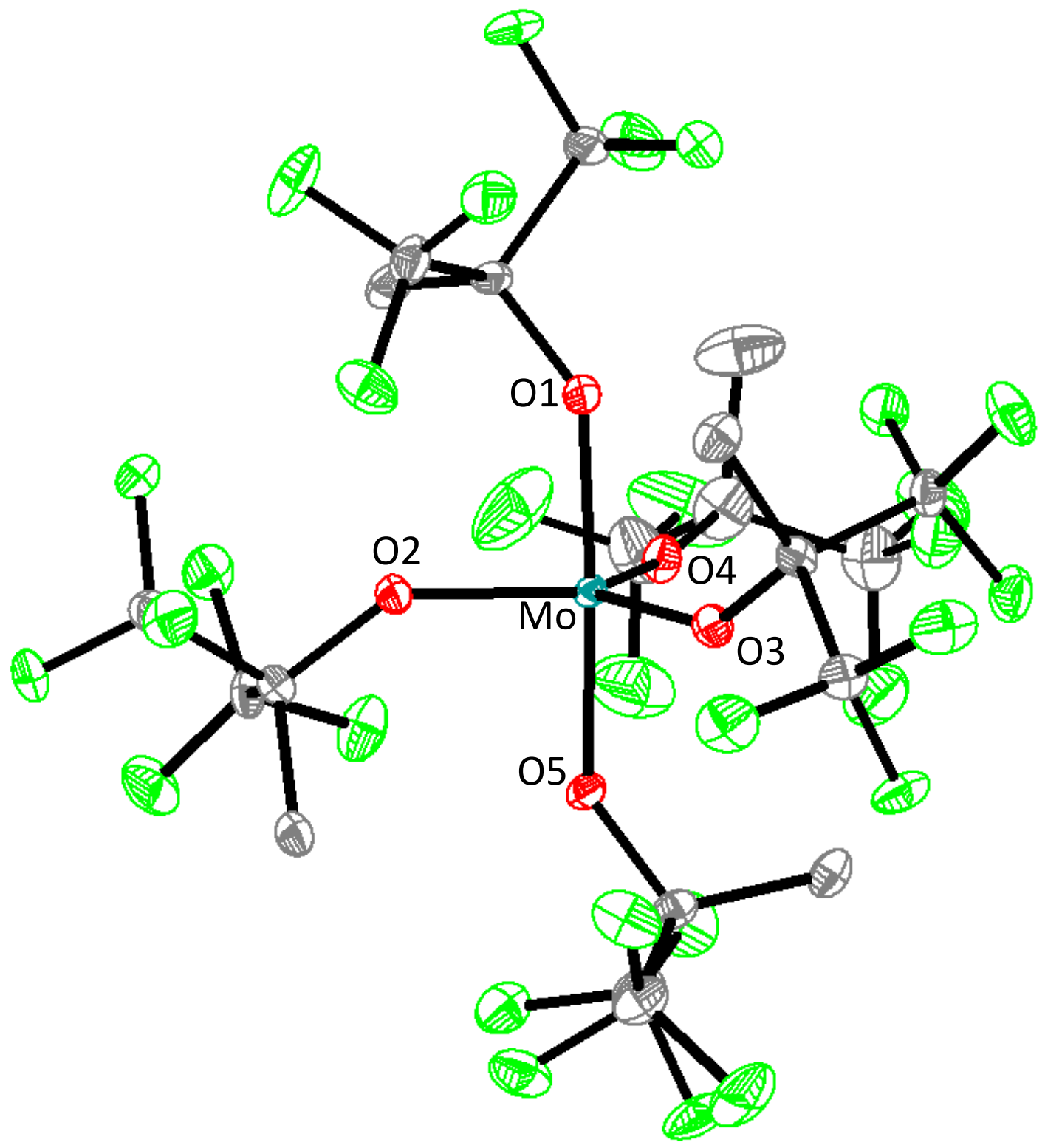

Figure S11. Solid state structure of $Mo(OR_{F6})_5$. Mo (teal), O (red), C (gray), F(green). C-H H-atoms omitted. CCDC 2522425.

Selected bond lengths (Å): Mo-O1: 1.942(4); Mo-O2: 1.885(4); Mo-O3: 1.893(4); Mo-O4: 1.833 (8); Mo-O5: 1.927(4); Selected bond angles (°): O1-Mo-O5: 171.8 (2); O2-Mo-O3: 120.1(2).

Table S2. SXRD structure of $Mo(OR_{F6})_5$. Notably, this complex has a Flack parameter of ~ 0, indicating a good fit for the absolute configuration assigned.

Empirical formula	$C_{20}H_{15}F_{30}MoO_5$
Formula weight	1001.26
Temperature/K	100.00(10)
Crystal system	orthorhombic
Space group	$Pca2_1$
a/Å	17.15930(10)
b/Å	17.92920(10)
c/Å	19.95420(10)
α/°	90
β/°	90
γ/°	90
Volume/$Å^3$	6138.96(6)
Z	8

ρ_{calc}g/cm^3	2.167
μ/mm^{-1}	5.586
F(000)	3896.0
Crystal size/mm^3	0.179 × 0.145 × 0.114
Radiation	Cu Kα (λ = 1.54184)
2Θ range for data collection/°	4.928 to 160.42
Index ranges	-21 ≤ h ≤ 21, -22 ≤ k ≤ 16, -25 ≤ l ≤ 25
Reflections collected	97112
Independent reflections	13145 [R_{int} = 0.0441, R_{sigma} = 0.0262]
Data/restraints/parameters	13145/1/1019
Goodness-of-fit on F^2	1.106
Final R indexes [I>=2σ (I)]	R_1 = 0.0494, wR_2 = 0.1464
Final R indexes [all data]	R_1 = 0.0533, wR_2 = 0.1514
Largest diff. peak/hole / e Å^{-3}	2.51/-1.05
Flack parameter	-0.031(4)

Computational Details

All calculations were performed using the ORCA quantum chemistry package (version 6.0.1), using default settings.[9]

Geometry Optimizations

Initial geometries were generated from the respective crystal structures. These structures were then refined with full DFT geometry optimizations using the B3LYP functional including Grimme's D3(BJ) dispersion correction.[10-13] The def2-TZVPD basis set was used for all atoms.[14] Harmonic vibrational frequency calculations confirmed all stationary points to be true minima (no imaginary frequencies).

EPR Parameters

Single-point calculations for EPR parameters were carried out at the B3LYP/X2C level of theory using the exact two-component (X2C) relativistic Hamiltonian. The X2C-TZVPall basis set was employed for all atoms, except for molybdenum (Mo), which was treated with the segmented all-electron relativistically contracted quadruple-zeta basis set x2c-QZVPPall-2c-s.[15] The resolution-of-identity approximation (RI-J) with the corresponding auxiliary basis set (X2C/J) was employed to accelerate Coulomb integral calculations.[16] Relativistic EPR and NMR parameters were computed using spin–orbit X2C formalism, including isotropic, dipolar, and orbital hyperfine contributions for Mo nuclei.[17] The chosen level of theory, B3LYP with X2C and relativistically optimized basis sets, has been validated in recent literature as a reliable approach for describing EPR parameters of Mo(V) complexes. These studies confirm that the X2C method accurately captures relativistic effects, and that B3LYP provides consistent trends and semi-quantitative agreement with experimental g-tensors and hyperfine couplings for d^1 Mo(V) systems.[18-20] Linear correlations between computed and experimental g-tensor components were then obtained by least-squares linear regression, performed in MATLAB (MathWorks, R2023b) using the polyfit function, and this was used to compute a corrected g-tensor for $Mo(OR_{F9})_5$. NMR shielding tensors were visualized using TensorView for MATLAB.[21]

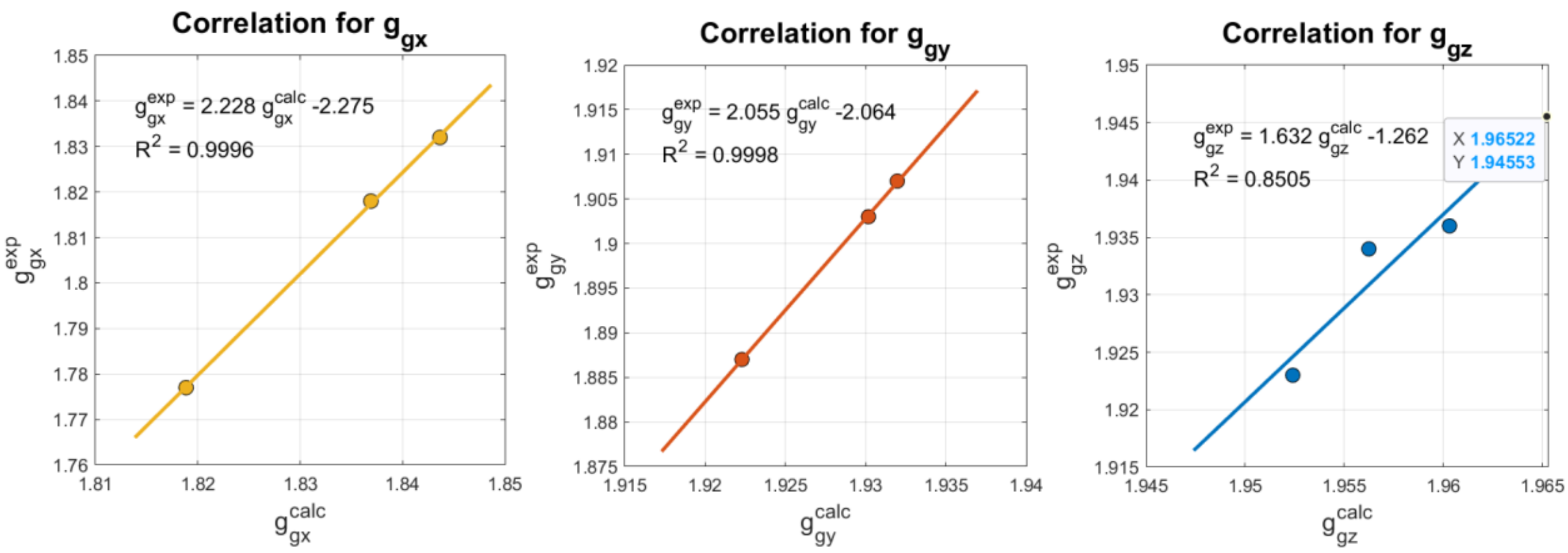

Figure S12. Linear regression fits of computed vs. experimental g-tensor components of Mo(O*t*Bu)$_5$, Mo(OR$_{F3}$)$_5$ and Mo(OR$_{F6}$)$_5$.

The magic pentagon used for qualitative EPR analysis is shown below.

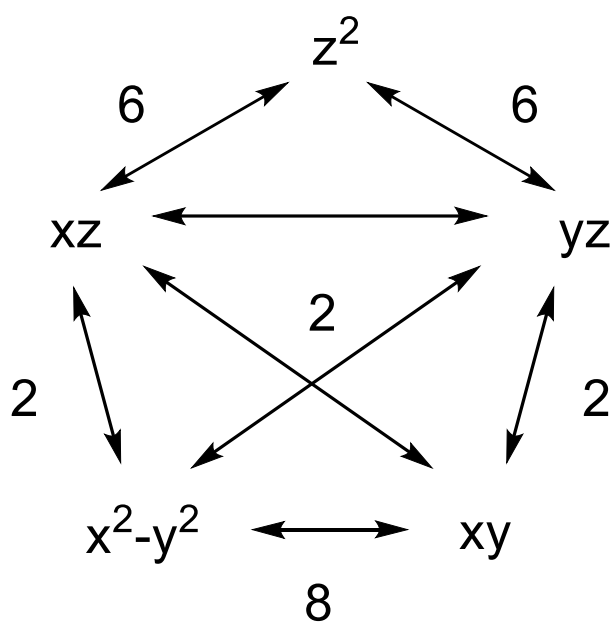

Scheme S1. Magic pentagon for EPR analysis, enabling qualitative comparison of electronic structure, d orbitals and energy gaps.

Assignments of d-orbitals were based on the xyz ordering of the g-tensor, in which the g_z component aligns with the z-axis defined by the axial ligands and the g_x component aligns with the longest Mo-O$_{eq}$ bond. Canonical orbitals from geometry optimizations are shown below

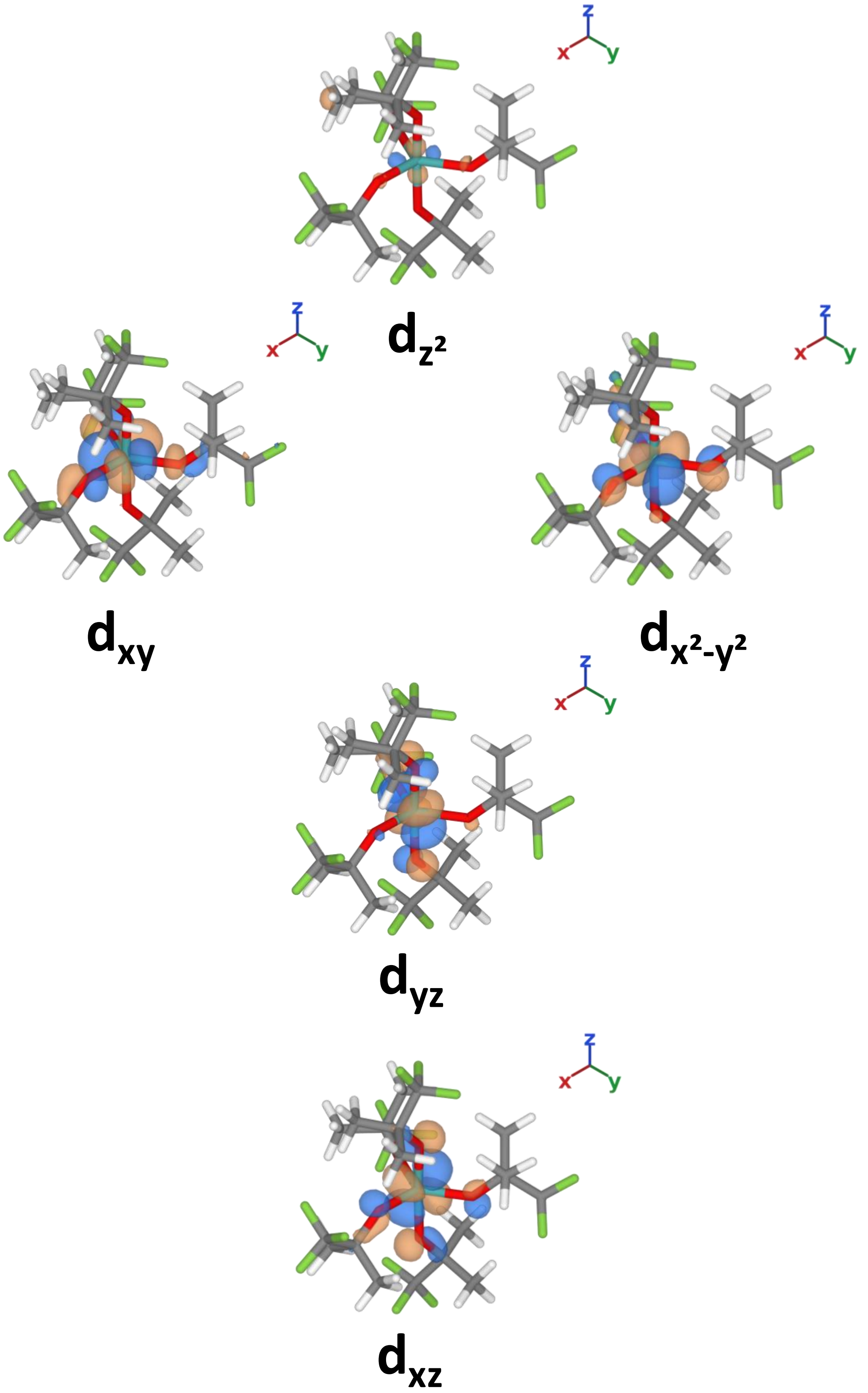

Figure S13. Canonical orbitals and assignments to d-orbitals. Z-axis defined by axial ligands, x-axis aligned with longest Mo-O_{eq} bond.

Population Analysis

Population analysis was carried out by generating all possible conformers from a GOAT-XTB calculation as implemented in ORCA. These structures were then optimized at the B3LYP/D3(BJ)/def2-TZVPD level, and the Freq, Hirshfeld and NBO keywords were used to invoke these calculations. Polarizability, quadrupolar moment and dipole moment were requested in the elprop block. The Gibbs free energy was used to generate Boltzmann weights for these properties.

Local Energy Decomposition

LED analyses were carried out at the DLPNO-CCSD(T)/def2-TZVP level of theory with def2-TZVP/C and def2/J auxiliary basis sets. The TightSCF keyword was used to ensure convergence of the Hartree-Fock reference. Fragmentation was defined as MoX_4^+ (cation, doublet) and X^- (anion, singlet).

Overlap Integral

The pairwise orbital overlap integral was computed numerically as the signed integral of the product of the donor and acceptor orbitals on the same grid. This was implemented by generating .cube files on the same grid (defined in %plots block in ORCA) for the HOMO and HOMO-1 of the isolated X-ligand, and the LUMO and LUMO+1 of the MoX_4^+ fragment at the B3LYP/def2-TZVP level of theory. Overlap integrals were numerically calculated using the Multiwfn 3.8 program by loading pairs of orbital cube files and applying grid product integration routine (13->11->6).[22] The absolute integrals were summed over the four donor-acceptor combinations to obtain a cumulative measure of orbital alignment.

$Mo(OtBu)_5$: MoX_4^+: 88 LUMO 89 LUMO+1		OtBu-: 19 HOMO-1 20 HOMO
19x88 Integral of all data:	0.0118008172	
20x88 Integral of all data:	0.0036433271	
19x89 Integral of all data:	0.0438071343	
20x89 Integral of all data:	0.1097319499	
$Mo(OtBu\text{-}F_3)_5$ MoX_4^+: 137 LUMO 138 LUMO+1		$OtBu\text{-}F3^-$: 31 HOMO-1 32 HOMO
137x31 Integral of all data:	-0.0818756558	
137x32 Integral of all data:	0.0845263876	
138x31 Integral of all data:	0.1049291507	
138x32 Integral of all data:	0.1141469979	
$Mo(OtBu\text{-}F_6)_5$ MoX_4^+ 185 LUMO 186 LUMO+1		$OtBu\text{-}F6^-$: 43 HOMO-1 44 HOMO
185x43 Integral of all data:	0.1088971133	
185x44 Integral of all data:	0.0252559586	
186x43 Integral of all data:	0.0387920417	
186x44 Integral of all data:	0.1406877764	
$Mo(OtBu\text{-}F_9)_5$ MoX_4^+ 233 LUMO 234 LUMO+1		$OtBu\text{-}F9^-$: 55 HOMO-1 56 HOMO
233x55 Integral of all data:	0.0051345946	
233x56 Integral of all data:	0.0963427501	
234x55 Integral of all data:	0.1218276485	
234x56 Integral of all data:	0.0365696172	

DFT Population Analyses

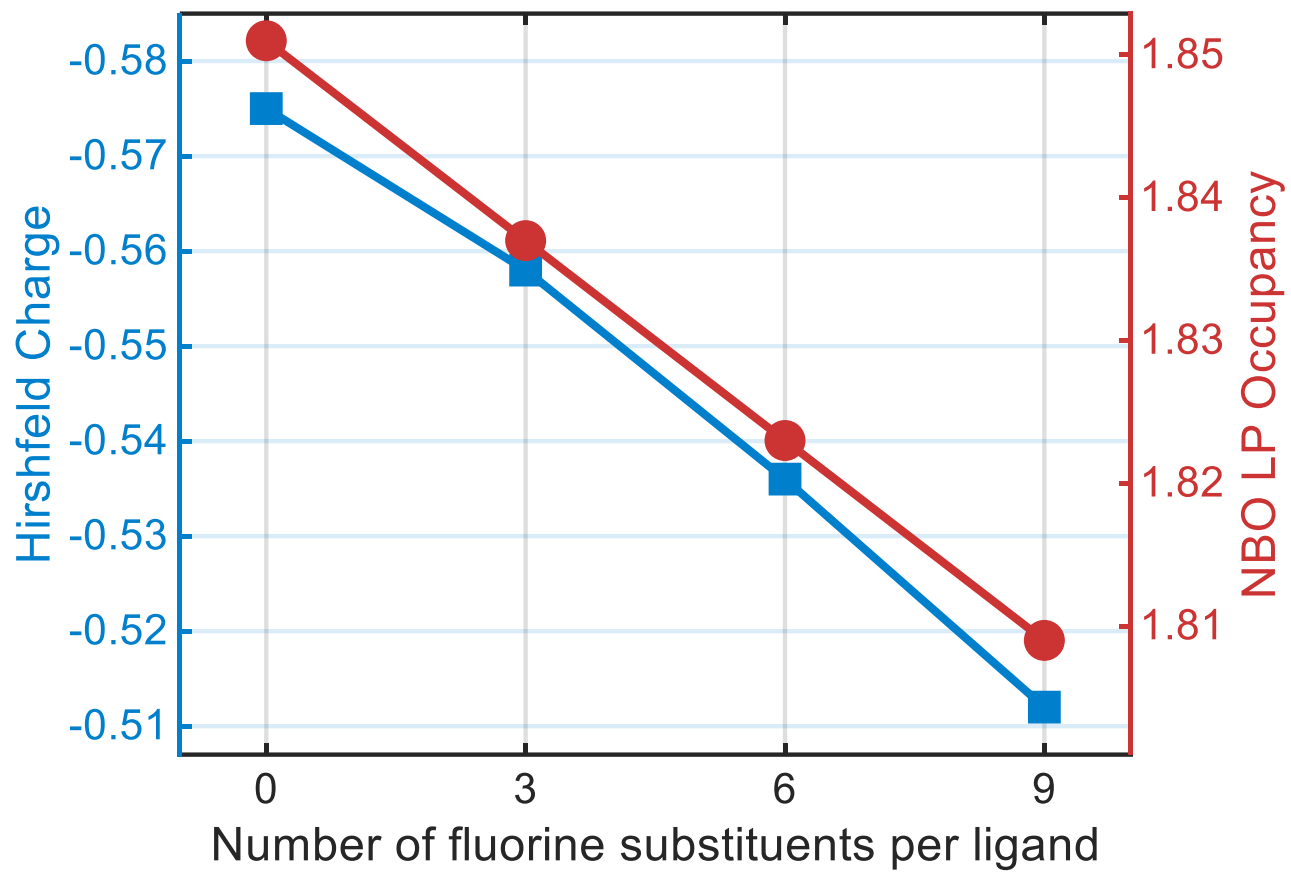

Figure S14. NBO and Hirshfeld population analysis for the optimized F_0-, F_3-, F_6-, and F_9-tertbutoxide fragments, calculated at the B3LYP/def2-TZVPD/D3BJ level of theory.

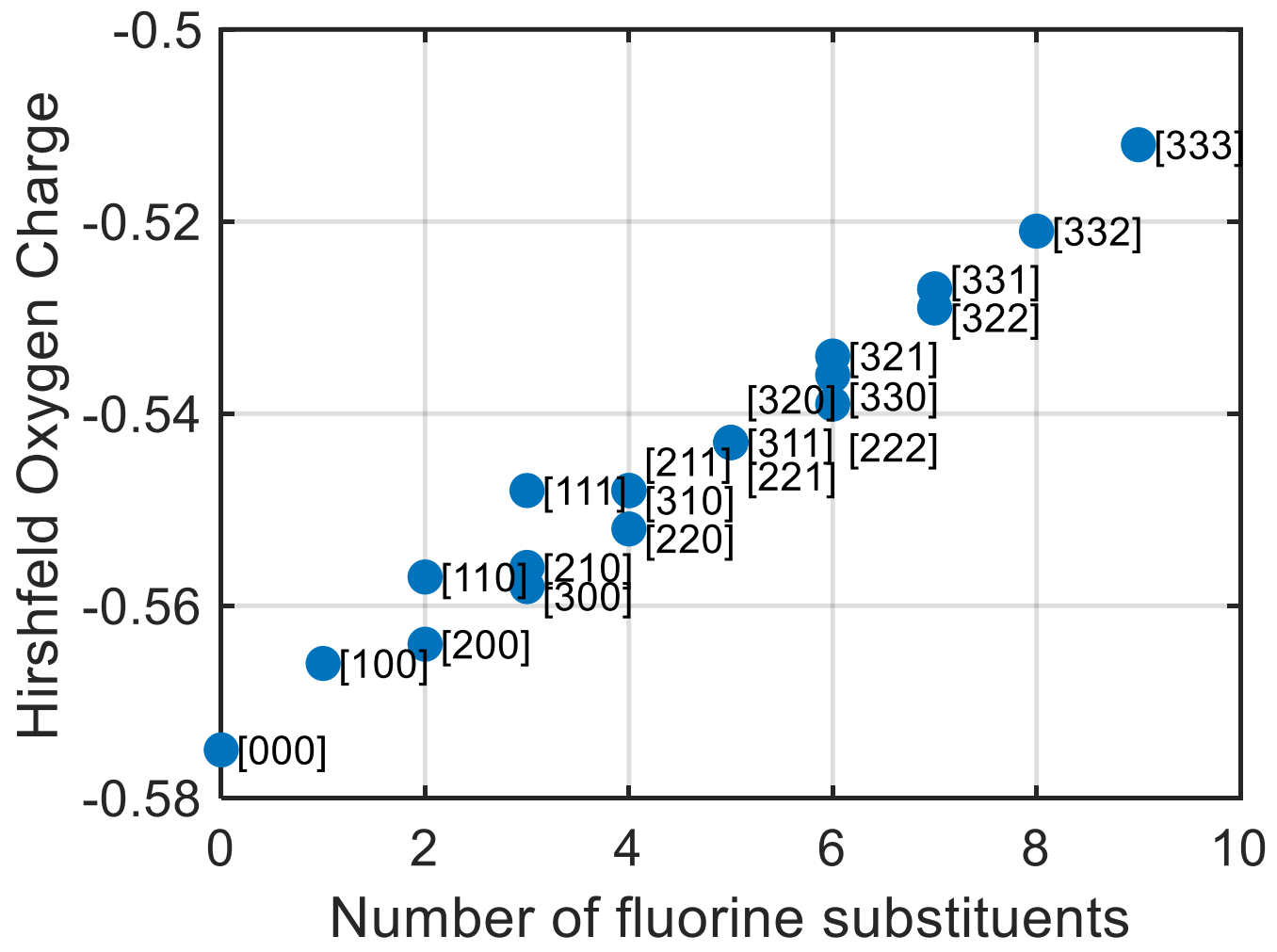

Figure S15. Boltzmann-averaged Hirshfeld charge on oxygen for all 20 isomers of non-fluorinated to perfluorinated tert-butoxide ligands. Index labels denote fluorine distribution on the ligand (*e.g.*, [111] = $(CFH_2)_3CO^-$).

Positional effects on fluorination

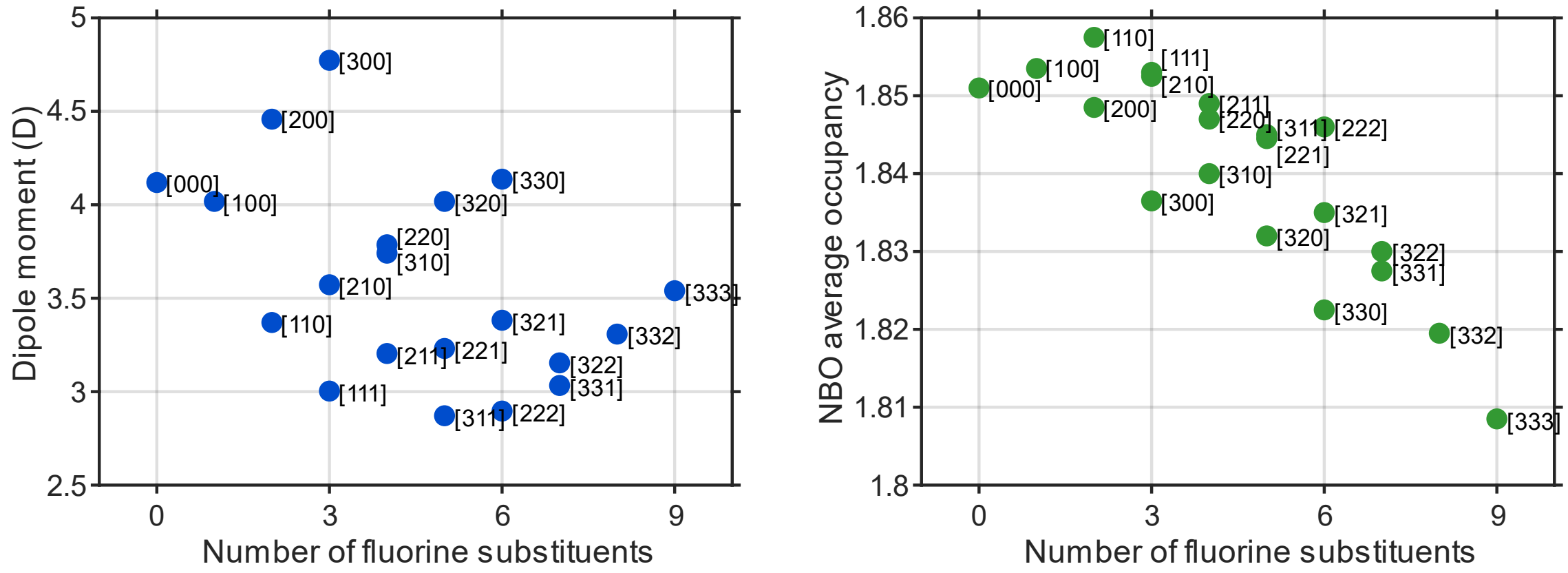

Figure S16. Boltzmann-averaged dipole moment for all 20 isomers of non-fluorinated to perfluorinated tert-butoxide ligands. Index labels denote fluorine distribution on the ligand (*e.g.*, [111] = $(CFH_2)_3CO^-$).Average NBO occupancy of the two valence lone pairs on oxygen across all 20 isomers of non-fluorinated to perfluorinated tert-butoxide ligands. Index labels denote fluorine distribution on the ligand (*e.g.*, [111] = $(CFH_2)_3CO^-$).

Looking forward, we were wondering if we could further tune the fluorination pattern in both the number and position to modulate donor strength. To comprehensively assess this, we systematically generated and optimized the complete set of 20 possible isomers from, covering all unique ways to distribute 1 to 9 fluorine substituents across the tert-butoxide ligand. Following a GOAT conformer search for the global minimum, we performed DFT calculations at the B3LYP/def2-TZVPD/d3 level of theory to investigate the dipole moment with Boltzmann averaging of the conformers (attached in the zip). The computed Hirshfeld charge on the oxygen atom increases linearly with fluorination, irrespective of position (Figure S14).

For each isomer we computed the gas-phase dipole moment as an intuitive descriptor for the directionality of charge separation within the ligand. Interestingly, the F_3 ligand seems to be the global maximum as well in terms of dipole moment (Figure S15). The two isomers with either one fluorine on all methyl groups F_3-[111], or one CF_2H and one CFH_2 group F_3-[210], both have a lower dipole moment and also less charge on oxygen. Introducing two fluorines on a tert-butoxide, the F_2 tert-butoxide, either increases F_2-[200] or lowers F_2-[110] the dipole compared to the parent tert-butoxide. We then analyzed the average NBO occupancy of both valence lone pairs for all the fluorinated isomers. This seems to confirm that the substitution pattern does matter, with the F_2-[110] isomer having the highest lone pair occupancy on oxygen. Synthesizing these 20 isomers of fluorinated tert-butoxide is outside the scope of this paper, and presumably comes with synthetic challenges related to the lower chemical stability of CH_2F and CHF_2 groups versus CF_3 groups. However, these results further reinforce the importance of spatial distribution of ligand substitution.

Table S3. Raw data for conformer contributions to alkoxide ligand parameters in the gas phase.

[nnn]	Avg Dipole (Debye)	Avg Hirshfeld Charge (O)	Avg NBO LP2 (O)	Avg NBO LP3 (O)	Conformer Contributions
0	4.119	-0.575	1.852	1.85	a: 0.497; b: 0.503
100	4.018	-0.566	1.864	1.843	a: 0.499; b: 0.500
110	3.37	-0.557	1.86	1.855	a: 0.443; b: 0.011; c: 0.449; d: 0.019; e: 0.010; f: 0.019; g: 0.011; h: 0.019; k: 0.019
111	3.003	-0.548	1.858	1.848	a: 0.328; b: 0.153; e: 0.167; g: 0.329
200	4.458	-0.564	1.859	1.838	a: 0.332; b: 0.338; c: 0.330
210	3.572	-0.556	1.857	1.848	a: 0.160; b: 0.161; c: 0.160; e: 0.151; f: 0.158; h: 0.013; i: 0.161; m: 0.013; p: 0.013
211	3.204	-0.548	1.854	1.844	a: 0.092; b: 0.051; c: 0.275; e: 0.035; f: 0.077; g: 0.094; h: 0.034; i: 0.080; j: 0.076; k: 0.051; l: 0.077; r: 0.034
220	3.786	-0.552	1.854	1.84	a: 0.400; b: 0.068; c: 0.068; d: 0.394; e: 0.022; j: 0.022
221	3.231	-0.543	1.851	1.838	a: 0.067; b: 0.443; c: 0.174; d: 0.174; e: 0.032; f: 0.034; g: 0.016; h: 0.017; q: 0.033
222	2.896	-0.539	1.847	1.845	a: 0.831; b: 0.059; c: 0.019; f: 0.018; i: 0.061
300	4.773	-0.558	1.853	1.82	a: 0.500; b: 0.500
310	3.741	-0.548	1.849	1.831	a: 0.340; b: 0.326; e: 0.329
311	2.871	-0.543	1.854	1.836	a: 0.134; b: 0.396; c: 0.019; d: 0.391; e: 0.020; g: 0.020; i: 0.020
320	4.018	-0.543	1.846	1.818	a: 0.297; b: 0.034; c: 0.035; d: 0.298; e: 0.297; f: 0.034
321	3.382	-0.534	1.843	1.827	a: 0.428; c: 0.010; d: 0.111; e: 0.056; f: 0.057; i: 0.108; l: 0.108; m: 0.106
322	3.154	-0.529	1.838	1.822	a: 0.406; b: 0.040; c: 0.039; d: 0.015; e: 0.025; f: 0.376; i: 0.014; j: 0.023; k: 0.041; l: 0.015
330	4.137	-0.536	1.836	1.809	a: 0.341; b: 0.338; c: 0.321
331	3.033	-0.527	1.836	1.819	a: 0.452; c: 0.538
332	3.308	-0.521	1.828	1.811	a: 0.258; b: 0.244; c: 0.232; e: 0.239
333	3.54	-0.512	1.809	1.808	a: 0.334; b: 0.329; c: 0.337

Conformer Analysis Schrock Catalyst

The initial transition state guess for the cycloreversion step was constructed from a previously published oxo analogue.[23] The TS was optimized using the r^2SCAN0 functional in combination with the def2-TZVP basis set with d4 dispersion corrections.[24,14,25] The optimized TS was confirmed by an IRC calculation using the same method.

Conformational searches were performed on both the TS and the GS using the GFN2-xTB method with the GOAT algorithm.[26,27] For the TS, the atoms forming the metallacyclobutane core were constrained to preserve the TS mode. The resulting conformers were re-optimized and subjected to frequency calculations to extract thermochemical data using the same method as for the TS optimization. To ensure that no conformer of the TS ensemble had lost the reactive mode, the Hessian of each conformer was inspected for the correct imaginary frequency. Gibbs free energies were computed using the quasi-Rigid-Rotor-Harmonic-Oscillator approximation at 298 K, as implemented in ORCA.

GS and TS conformational ensembles were analysed using the ASE package.[28] Boltzmann populations were calculated from the Gibbs free energies relative to the lowest-energy conformer within each ensemble and assigned to the corresponding dihedral angles of each conformer. The resulting weighted dihedral distributions were binned in 15-degree intervals and displayed as polar plots (Figure 7c). The GS conformer structurally closest to the lowest free-energy TS conformer was identified by RMSD after optimal superposition, and is highlighted in the energy diagram with a dashed connector (Figure 7b). Full analysis code is provided as script in the supporting .zip file.

Rotated TS conformers were selected from the optimized TS ensemble based on the Mo-O-C-CH_3 dihedral angles θ1 and θ2. For each rotation pattern, the conformer with the smallest joint angular deviation from the idealized 120° rotations of the two alkoxide ligands was selected. Because the final TS optimizations were performed without dihedral constraints, the optimized geometries do not correspond to exact 120° rotations. This procedure was preferred over a constrained dihedral scan because each selected structure is a fully relaxed first-order saddle point on the cycloreversion potential-energy surface. The resulting relative Gibbs free energies therefore describe accessible TS conformations without the artificial strain associated with fixing the alkoxide dihedrals at prescribed values.

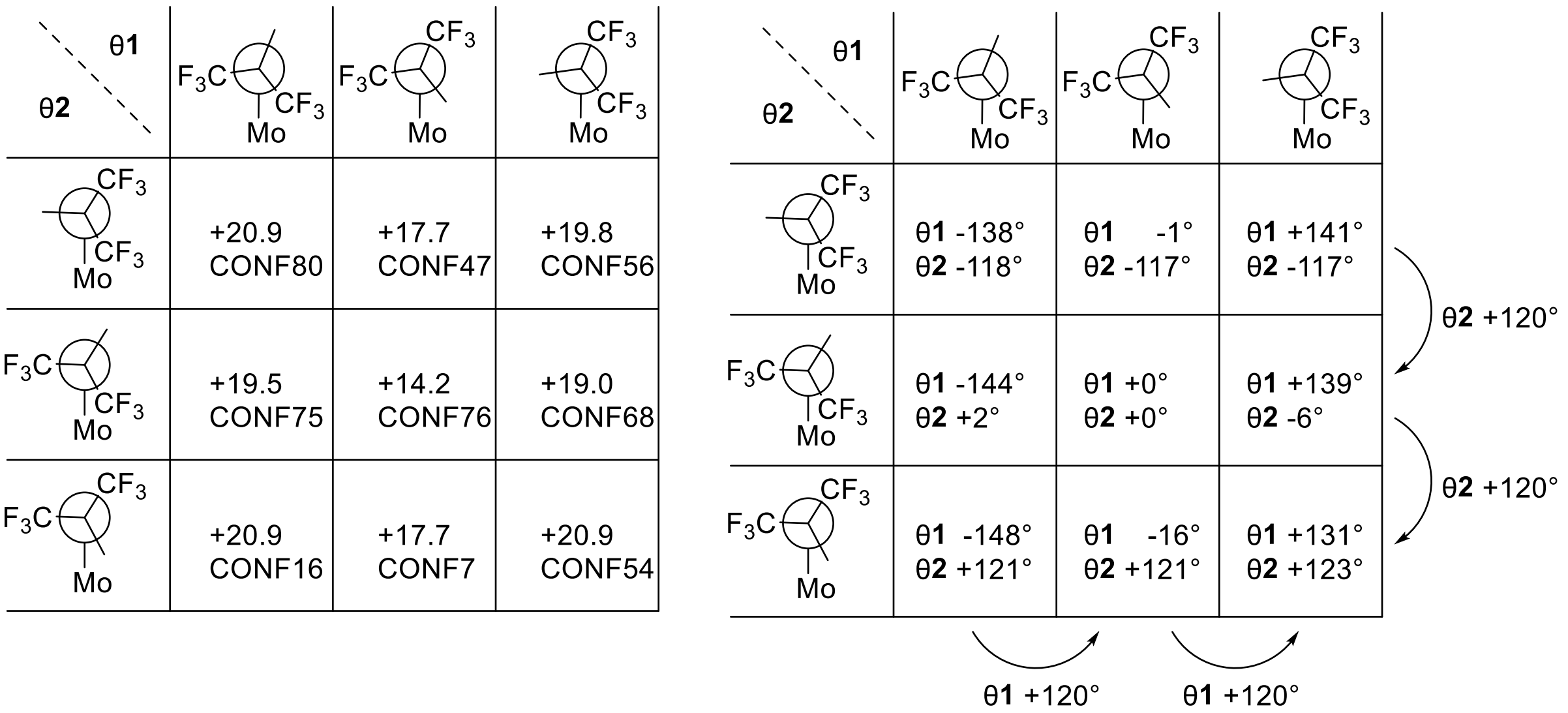

Figure S17. a) Diagram showing assignment the transition state conformers discussed in Figure 8 to conformer indices used in repository dataset at 10.5281/zenodo.20569296. b) diagram showing actual changes in dihedral angles 1 and 2.

Coordinates of Calculated Structures

Mo(OtBu)$_5$

C	8.61207336811501	8.66815156105848	4.31351882785681
C	9.30260455315668	10.02680865567551	4.33883219829625
H	8.56913791624532	10.81913071614628	4.49526316818326
H	9.81495990515196	10.20894838284655	3.39199354445500
C	9.62750427087295	7.53226086721492	4.19496481022625
H	9.11731746083454	6.56707676664376	4.21471131089258
H	10.33914568858593	7.57294687466934	5.01697188321927
H	10.17260747739919	7.61036219023339	3.25243801654622
C	9.49316628054356	5.23752969178662	8.16055642893570
C	9.65791752197293	5.40062057653322	9.66846290118232
H	10.32986993118551	4.63644619860391	10.06292119428360
H	8.69057068265860	5.30299286757035	10.16262818223647
H	10.06898836151467	6.38029997190962	9.90487607059538
C	8.86480939980498	3.88768770004918	7.82245960084922
H	8.70466634065678	3.80342777316712	6.74709678588723
H	7.90404031104092	3.77850817384418	8.32665295030357
H	9.52047772808799	3.07503154986931	8.14026522304173
C	10.81006475641110	5.42827049472377	7.41761244384314
H	11.20163671360337	6.42798286460984	7.58371774710924
H	10.66290207864028	5.29477749859213	6.34530128233351
H	11.54173349686774	4.69499627301639	7.76140370630638
C	10.88833837326229	9.43272772287702	7.87137582832787
C	12.20637510821940	9.33991598444164	7.09618713875446
H	12.56017369592547	8.30813919290640	7.06819944440233
H	12.97668914311682	9.95541589525816	7.56560310087420
H	12.07370354686684	9.68113904722513	6.06875962486353
C	10.38376452247061	10.88010911794731	7.87168275468460
H	11.11806856834369	11.53157202015558	8.35021069200529
H	9.44405796940685	10.94990382766362	8.41161330353794
H	10.22704592733907	11.23602806070134	6.85416684841655
C	11.09982364765165	8.95362655178895	9.31091509657252
H	10.15068315923448	8.93778190334722	9.84197027841833
H	11.78635184255247	9.61954624082087	9.83776190587892
H	11.53055730362104	7.95184312302124	9.31993274433233
Mo	8.10980929427871	7.96698997211998	7.30493004549783
O	7.83450259200379	8.50546123126751	5.51359834433736
O	8.54833157950193	6.22749807118932	7.72263650969688
O	9.95848802278126	8.57954102125022	7.20698092125436
H	10.03167265495975	10.05942133620651	5.14163127059687
C	7.60488873415449	8.60508681299096	3.16442063643285
H	6.86183599356433	9.39690306667936	3.26686863440693
H	7.08726560612329	7.64541345387489	3.15847448019195
H	8.11367494488336	8.72949169638972	2.20652811556563
C	6.86712462720791	9.54080218282059	9.67655073725574
C	5.76597359236748	10.27442665643944	8.91445430655827
H	5.00507856423152	10.63927485714905	9.60765022986584
H	5.29965813968426	9.61159909712006	8.19085189689442
H	6.18795121244441	11.13142031102549	8.38621036638511
C	6.33671730867246	8.30299630221746	10.39790810095741
H	7.15686964876261	7.78556754847109	10.89984662162633
H	5.86969184922079	7.62176637810279	9.69203147726142
H	5.60241732319497	8.59296037481438	11.15224611580456
C	7.53099002289379	10.48631592894581	10.68046725306952
H	7.93350229239853	11.36385428493206	10.17355542715584
H	8.34802461783381	9.98244371780549	11.19854040052279
H	6.80462992829998	10.82066105999889	11.42374862349281
C	5.25559549080415	6.91718157526156	6.56566982507475
C	4.23687769544049	6.30766642066004	7.53230136624780
H	4.73068005640122	5.59980199441886	8.20025722181349
H	3.44972757226125	5.78070893114952	6.98877157158835
H	3.77686815536628	7.08938948740491	8.13843742234446
C	4.58029137985488	7.94684323782684	5.65660543935267
H	3.82555724982874	7.46856481047759	5.02829435307011
H	5.32092397737979	8.42492345117383	5.01936615964498
H	4.09138847816546	8.71597747976731	6.25647364212217
C	5.91195123465715	5.80938297592699	5.73715220458305
H	6.65299409797827	6.22634347678596	5.05720994496817
H	5.16073878093642	5.28112318442117	5.14663865070461
H	6.40660319767183	5.09131133330597	6.39053558965782
O	7.92192120376327	9.16483141033122	8.76573679154056
O	6.21418816766559	7.59683302035783	7.37006157080341

Mo(OtBu-F$_3$)$_5$

Mo	3.67293583844987	7.99701516009099	3.01538624948428
F	0.61291168630324	8.84070864448122	6.36150390969298
F	1.72917877890915	8.28594873343771	8.13500190102010
F	1.24627269091058	6.79160078851350	6.63976619700976
F	0.89498974112063	10.68993270854618	0.57997703039054
F	-1.03144205467584	9.91987796335142	1.20986887166252
F	0.37246803858374	8.62550812819603	0.18371737786624
F	6.43798819075436	10.76907239492995	5.39461709764013
F	7.07711476134469	8.74152370365536	5.81011380423942
F	8.36633878973014	10.07614750212622	4.68952870347476
F	2.48112437685639	4.20831049087755	1.48928089966646
F	3.47873739536441	2.64511371130195	2.61910854995753
F	4.64170021420840	4.06369827364615	1.45867702539831
F	6.15937763423085	6.52732542696057	-0.15833783158369
F	6.67204776461987	8.61342359464764	-0.42939059887767
F	5.61404967161995	7.54850154158439	-1.99472712032081
O	2.73403595789397	8.09486702770334	4.72373039964287
O	2.23352299173048	8.91149167765870	2.18438024429055
O	5.11980196649349	8.85654007563353	3.90718403464624
O	3.74267991382560	6.16757357286428	2.95368624423813
O	4.61438152583355	8.20763336267460	1.34848449124048
C	2.96206618526230	8.38736861088077	6.08594820390175
C	1.62989629217001	8.07587288520249	6.80638021898369
C	3.29158488246852	9.86735250807573	6.29141223995881
H	4.22074217094991	10.09641025757825	5.78312504884164
H	2.50558180393067	10.50152109000498	5.88808065368305
H	3.40606338437846	10.08745525601457	7.35227384802004
C	4.05321591124505	7.49159297122894	6.67488606201361
H	3.77001163394564	6.44251570316253	6.61032863526964
H	4.97369046214635	7.65289309047177	6.12294446844348
H	4.22840229965510	7.73501462359225	7.72199461462093
C	0.86663145817227	9.20105357262022	2.46585362922268
C	0.27671401605787	9.61150585569746	1.09707123218890
C	0.13080951149402	7.96102406019567	2.96814781320564
H	0.25473924212880	7.13720711976648	2.26542220012267
H	0.53681723262872	7.67491908593713	3.93295015138882
H	-0.93266477945894	8.16728642428501	3.08130248412836
C	0.75534460490058	10.38537565606015	3.41789050458638
H	1.12902004395542	10.09026717928918	4.39008031646461
H	1.33811892598611	11.22622756445410	3.04475948065648
H	-0.28385862621363	10.69388776072408	3.52646227769568
C	6.44941911439040	9.23428700928527	3.54054002485468
C	7.08205653785312	9.70852602846831	4.86939142777901
C	7.23930263386444	8.03056672287653	3.03191988404816
H	7.22290147518299	7.22885247625344	3.76988515485994
H	6.79621514024853	7.67453156200841	2.10889010811118
H	8.27507367984332	8.30692691606526	2.83928622991996
C	6.44227654500198	10.40501147270985	2.56222744760272
H	6.04485856405936	10.07050859655664	1.61127530627979
H	5.82319377655226	11.21403315712618	2.94804666991296
H	7.45441172040095	10.77815296120414	2.40888777334407
C	3.63435433404664	4.85489087182003	3.48520958413129
C	3.56112322448332	3.93744656238776	2.24452768569073
C	2.35912306265187	4.71614949127859	4.30307010144898
H	2.38331720212142	5.41148074953906	5.13502263167947
H	1.48548145293845	4.93982008338163	3.69344305736378
H	2.27012019427420	3.70189503645084	4.69033443364248
C	4.89709651439226	4.54844609954915	4.28119793005196
H	5.78039523777250	4.68223484394991	3.65907140348888
H	4.95699384576131	5.22867738695432	5.12775060049729
H	4.87479343415861	3.52529189344288	4.65396322804802
C	4.35503577245078	8.10373369604924	-0.03726334116737
C	5.70892977215364	7.69525077046088	-0.65710839093501
C	3.32456326753897	7.02035648044949	-0.34834243604687
H	3.64771123089239	6.05850782050593	0.04005872967584
H	2.37616348626356	7.29020338465881	0.10720399701922
H	3.17451090152455	6.93260433079964	-1.42371211715776
C	3.93956232145870	9.46698377213323	-0.58942232607572
H	3.01419661516124	9.77059149559870	-0.11192689820763
H	4.70237993329943	10.21591277954578	-0.38306932566249
H	3.78144347934774	9.41240379036634	-1.66620713837383

Mo(OtBu-F6)5

C	12.72626560597903	13.39899911454756	5.05216173676452
C	11.52569547313150	14.30267816798302	4.83131715917984
H	10.67088655348937	13.91212139517414	5.37642775337557
H	11.28654405034194	14.35496310122654	3.77146588200299
H	11.74666212864870	15.29903553198363	5.19046796899395
C	12.43725331569878	11.99861410956844	4.43667574943056
C	14.01037463612335	14.04182490314788	4.45436821628945
C	14.46216564523349	10.60983727964359	8.42328821234364
C	13.32323331066327	9.83720867906967	7.76239403822787
H	13.09549640181939	10.29064129172178	6.80354556479505
H	12.44198911487320	9.86658018673244	8.39118280628087
H	13.60590220437607	8.79981312389587	7.59873520008807
C	14.68679791040467	10.06939186960040	9.87176753860443
C	15.76023059990345	10.42424135901623	7.58048621421789
C	10.37579063597938	12.02685561842706	9.60876485983213
C	9.73232061452154	13.25220172755042	10.23881955540006
H	9.53425214262198	13.99935682971913	9.48322763204683
H	10.40525741493265	13.66221218804198	10.98640554006862
H	8.79448942477960	12.97711538716854	10.71657105184993
C	9.56746992000761	11.58730031660286	8.35520882298183
C	10.42747274749932	10.88823763978413	10.67268186130226
C	11.33689432384228	16.11490748779906	8.33863845655083
C	12.46660191122797	16.93582461437452	7.71619807254909
H	12.67641528010718	16.55236876531173	6.72197773257501
H	13.36225140167631	16.85248426400490	8.31638854752376
H	12.18890996396985	17.98486630683523	7.63941558858793
C	11.17674057667427	16.49065517847085	9.85925986471424
C	9.99808458677101	16.48439325416040	7.61984845367639
C	15.31472966490304	14.74280648613302	9.61904596512121
C	15.91016341155083	13.53239863329252	10.32847841054889
H	16.22817440656192	12.78955757771938	9.61180608865209
H	15.16283701549752	13.09806797244622	10.98680522343726
H	16.76830477410843	13.84084276877187	10.92216547724413
C	16.19600862411512	15.13018366269697	8.39778472158838
C	15.23878220488102	15.91173333770340	10.64867566268191
F	12.12284149631591	12.10314027182893	3.13878182229007
F	13.46631494505736	11.15622416770881	4.54582836333185
F	11.39068503655771	11.44691988937271	5.07430531974175
F	13.86780967978118	14.25927354016383	3.13941704972582
F	15.09556620675285	13.29414676000038	4.64418131769162
F	14.22337885169441	15.23042794814813	5.04862055121135
F	14.62769376303143	8.72944984029175	9.91037231724524
F	15.87207156182669	10.43164171884217	10.39066442406872
F	13.73573580928030	10.54464977250910	10.68794305627572
F	16.77654009672097	11.18407125651403	8.01041117016232
F	16.16946310924661	9.14347819052446	7.59859750230944
F	15.52702482333412	10.76231450824748	6.30449202973945
F	9.67255992608845	12.53245085857001	7.40233709220345
F	10.01640395064500	10.44208970162996	7.82345513292996
F	8.26838448467467	11.43274314140293	8.63575935887698
F	11.12605319720270	11.30507525133199	11.73757089588857
F	11.00477123338914	9.76975597915767	10.21792282052866
F	9.19246807925717	10.56442820689907	11.08983534686888
F	11.98415669026011	15.74621462872907	10.62265966411585
F	9.92400026899749	16.29692672843451	10.31365375129230
F	11.47970195152660	17.77807302227114	10.07587183054009
F	9.01041392365298	15.62031824277138	7.88315861570334
F	10.15683234976909	16.49786450188047	6.28276793223710
F	9.58679878401072	17.71345958188106	7.97453006151129
F	16.07601708791298	14.18961392778256	7.44691142660029
F	17.49013256646784	15.21997257499281	8.72605409997153
F	15.82631239073450	16.29883841412193	7.84895414562499
F	16.46772605401652	16.28089169260647	11.04592500126043
F	14.62804012234230	17.00303497605369	10.16937081079205
F	14.56037000977270	15.51220040037841	11.73408221689288

Mo	12.88558286017947	13.33364798333352	8.24177313229156
O	12.99835364723461	13.19615089513627	6.41395384234589
O	14.24692658803483	11.98585342444874	8.50468575852234
O	11.71085230726821	12.21281987485616	9.20412082213963
O	11.51860544213668	14.73884410989692	8.20526299544408
O	13.99982270790934	14.58785688695469	9.13069469209619

Mo(OtBu-F9)5

C	8.55269429936875	8.94854064356557	4.26591266514114
C	8.98550625768995	10.46106968154487	4.15246703195964
F	8.18647487491577	11.21341329169384	4.90655263245719
F	8.89681200089489	10.88456057043505	2.88716656365191
C	9.70985560037604	7.99474901067157	3.79421415802546
F	9.22298555064935	6.78117119908809	3.50837297810830
F	10.60620053572966	7.84835853607711	4.76585956760906
F	10.32500661192175	8.47292195616272	2.71321203838434
C	9.35505651643098	5.11151269385910	8.33938031460825
C	9.56309348267539	5.18565490750895	9.89343505130794
F	10.52238643535354	4.34482237627191	10.28714933498235
F	8.43470937582016	4.87406039282528	10.53686815580652
F	9.90220026491016	6.42167243909935	10.24773118429156
C	8.56612811131267	3.79485108461253	7.98827173563530
F	8.58274088207125	3.59116713851895	6.66644167581899
F	7.30600107690894	3.88285222589687	8.38755478903576
F	9.11866343183209	2.73570851903738	8.58800632072318
C	10.73523731277277	5.08019422002350	7.58283906441080
F	11.64090994409948	5.81839265061806	8.21205813560556
F	10.57034315160564	5.56170663583993	6.34640255529597
F	11.21561658472741	3.83805834939175	7.48770627833498
C	11.11913973351793	9.31962178784777	8.00119022325355
C	12.37093106494687	9.12718644646186	7.04816173972516
F	12.57983000603180	7.83432113435691	6.80829914193774
F	13.48346357669730	9.63364000286530	7.59374043854378
F	12.18112194035804	9.73112569871715	5.87281665851582
C	10.65280626239005	10.83645758255626	7.94975948351808
F	10.22504194699579	11.26966284873052	9.13498475746536
F	9.63818099294293	10.96836158550190	7.09127844611741
F	11.64135755952480	11.64963120001946	7.56513610417725
C	11.59000214256666	9.01546029683116	9.47716203475398
F	10.53894727981194	8.87534288519397	10.27963305428253
F	12.35214231100012	10.01058162416065	9.95030233484557
F	12.31268704863226	7.89453201914988	9.53529700604247
Mo	8.24727485239246	7.93767766924847	7.32087991080984
O	8.21664341924270	8.64814802228937	5.58282821040463
O	8.59366412028710	6.20339546937540	7.94307390147854
O	10.12638766442851	8.45220033389326	7.59954356715735
F	10.24333158724413	10.63381596958695	4.54620098492411
C	7.31172301507905	8.75245779987309	3.30574083367892
F	6.46636208334488	9.77824585187678	3.40039693264037
F	6.65346278373303	7.63621022920975	3.59964942390323
F	7.72044504208874	8.66338966024738	2.03579625265714
C	6.82993458624197	9.63083252018616	9.54909683563434
C	5.67000658952090	10.23545683841939	8.66552175190561
F	5.05615161037198	11.25114536955415	9.27721451097022
F	4.74133518872391	9.32843396320542	8.38110401252896
F	6.18766271790355	10.68786313652974	7.51767814698614
C	6.30786024416304	8.51637553055353	10.52905188342911
F	7.15763661793885	8.34982780764534	11.54784131650193
F	6.21499466517988	7.34350183388874	9.90194453484277
F	5.11155613838105	8.83521698013783	11.02706687431480
C	7.42881051740877	10.80829958729760	10.41497175923577
F	7.65180585959052	11.87567414847752	9.64138543876691
F	8.57079058177855	10.45578239955542	10.98825634433082
F	6.57337184398367	11.15772980704682	11.38415931703759
C	5.36238111586172	6.73945729206974	6.53366060699961
C	4.45350507779074	6.03902964448919	7.62608794079097
F	5.16264116719045	5.21076228307597	8.38947565273956
F	3.49163147658889	5.34223598371348	6.99429098152334
F	3.87025089853329	6.90032830513668	8.45376746990897
C	4.43791125529655	7.79475406292774	5.72497862312943
F	3.98258399710968	7.34241213538900	4.55761337950729
F	5.15298973572008	8.89810352261986	5.49845224628036
F	3.36077181300856	8.15551931288126	6.42769289450970
C	5.97858553633855	5.57477445099026	5.59560216616840
F	7.28997583890617	5.82596308511261	5.41032682986480
F	5.42169275252988	5.45291632882370	4.39668134121754
F	5.89031970904658	4.37541618359014	6.17239778504733
O	7.86639674830023	9.13086400528879	8.76037241572926
O	6.36010932026584	7.44720332865604	7.15850257406943

OtBu-

O	-0.66177253562429	0.43448513842367	2.80842221849092
C	-0.54799783883437	0.40316078433357	1.47355104451738
C	-1.52063203702414	1.41648972590008	0.78342603233297
H	-1.29601198667656	2.42230850313700	1.15049642423806
H	-1.45803693574344	1.41801647001340	-0.31522655800100
H	-2.54571400685031	1.17267325938756	1.07763766426937
C	-0.87572200809635	-1.01380291018554	0.89580008554921
H	-0.79124732191677	-1.08066238550220	-0.19938908759079
H	-0.19403586481714	-1.74110949379878	1.34647235037514
H	-1.89515278100435	-1.28344038256727	1.18727287764332
C	0.90036066785224	0.76627787321958	1.00506690388723
H	1.60150863812510	0.05615176429241	1.45330694010502
H	1.14799076069830	1.76427921383063	1.37842219813930
H	1.03235052801004	0.75332821285727	-0.08727871141625

OtBu-F3-

O	-0.63755565903768	0.40997254616094	2.82162910528335
C	-0.51300159935620	0.36669633081343	1.50180921766059
C	-1.49429513649735	1.38720912840295	0.83043771066943
F	-1.26507136335776	2.66864082679502	1.21019456612817
F	-1.43981142019233	1.41425079469510	-0.55325148132191
F	-2.79539344446009	1.13698375226142	1.12001789026894
C	-0.86540701121757	-1.03314021207557	0.89920282154645
H	-0.79543843696366	-1.08572558682163	-0.19407497480116
H	-0.16589569327622	-1.74814293446312	1.33715885601438
H	-1.87366676880931	-1.31107238444108	1.21102793109612
C	0.91973532964237	0.75577472376991	1.00908733668841
H	1.60913898725452	0.02850573167183	1.44293686411439
H	1.17457007012821	1.74104982909781	1.40300561636714
H	1.03797942424104	0.75715322747436	-0.08120107717445

OtBu-F6-

O	-0.65438910041069	0.43758012970902	2.81678428736276
C	-0.51749233191431	0.41330952094061	1.51464233454742
C	-1.50184238130239	1.44237930096092	0.82571260699239
F	-1.22089253382976	2.70120630976602	1.24027769872612
F	-1.44354361122835	1.49519449039138	-0.54559307875401
F	-2.79927238894988	1.22466950978230	1.12532250107564
C	-0.84909773720075	-1.02316388650031	0.94103931092305
F	-0.75162119997194	-1.15722336721192	-0.42247114955294
F	0.01404785785848	-1.93598029133827	1.44870028149847
F	-2.08725096114302	-1.45327247936721	1.26044483586269
C	0.91693687393095	0.76987249841282	1.00352895780088
H	1.60761433770457	0.06009367146358	1.45744550340978
H	1.15419626408473	1.76562602465209	1.37649503414264
H	1.02849419047032	0.74786434168034	-0.08434874149505

OtBu-F9-

O	-0.66279685238293	0.43494772156141	2.81824294540790
C	-0.55327032014493	0.40475408177478	1.53362902261567
C	-1.51459464289834	1.46667268173755	0.82076531253092
F	-1.47356454019003	2.64847657111771	1.45624063111073
F	-1.23803771827159	1.72832948853267	-0.48873093700369
F	-2.80335732939347	1.05539706332166	0.85622931363977
C	-0.92612569076320	-1.02912715268538	0.92947445957725
F	-1.14882327047221	-1.06449437873868	-0.41526585676875
F	0.05683553405816	-1.92768293959809	1.16993310924800
F	-2.03657408891263	-1.51565648890318	1.50633175045838
C	0.93478881480549	0.73426898094014	1.04656892579318
F	1.83724339862173	0.08486060462328	1.79914436379593
F	1.20676885650831	2.05368812822800	1.17381765864815
F	1.21739512753362	0.41372141142951	-0.24840031651357

References

1 Nater, D. F.; Kaul, C. J.; Lätsch, L.; Tsurugi, H.; Mashima, K.; Copéret, C. Olefin Metathesis Catalysts Generated *In Situ* from Molybdenum(VI)-Oxo Complexes by Tuning Pendant Ligands. *Chem. Eur. J.* **2022**, *28* (22). https://doi.org/10.1002/chem.202200559.

2 Favero, L.; Marchetti, F.; Pampaloni, G.; Zacchini, S. The Interaction of Molybdenum Pentachloride with O- and S-Heterocycles. *Dalton Trans.* **2014**, *43* (2), 495–504. https://doi.org/10.1039/c3dt52271g.

3 Stoll, S.; Schweiger, A. EasySpin, a Comprehensive Software Package for Spectral Simulation and Analysis in EPR. *J. Magn. Reson.* **2006**, *178*, 42–55. https://doi.org/10.1016/j.jmr.2005.08.013.

4 Casey, T. *cwEPR*, Version 3.6.0; MATLAB Central File Exchange: 2024. https://www.mathworks.com/matlabcentral/fileexchange/73292-cwepr (accessed May 8, 2025).

5 Sheldrick, G. A Short History of SHELX. *Acta Crystallogr., Sect. A: Found. Crystallogr.* **2008**, *64*, 112–122. https://doi.org/10.1107/S0108767307043930.

6 Sheldrick, G. SHELXT—Integrated Space-Group and C rystal-Structure Determination. *Acta Crystallogr., Sect. A: Found. Crystallogr.* **2015**, *71*, 3–8. https://doi.org/10.1107/S2053273314026370.

7 Sheldrick, G. Crystal Structure Refinement with SHELXL. *Acta Crystallogr., Sect. C: Struct. Chem.* **2015**, *71*, 3–8. https://doi.org/10.1107/S2053229614024218.

8 Dolomanov, O. V.; Bourhis, L. J.; Gildea, R. J.; Howard, J. A. K.; Puschmann, H. OLEX2: A Complete Structure Solution, Refinement and Analysis Program. *J. Appl. Crystallogr.* **2009**, *42*, 339–341. https://doi.org/10.1107/S0021889808042726.

9 Neese, F. Software Update: The ORCA Program System, Version 5.0. *WIREs Comput. Mol. Sci.* **2022**, *12*, e1606. https://doi.org/10.1002/wcms.1606.

10 Becke, A. D. Density-Functional Exchange-Energy Approximation with Correct Asymptotic Behavior. *Phys. Rev. A* **1988**, *38*, 3098–3100. https://doi.org/10.1103/PhysRevA.38.3098.

11 Lee, C.; Yang, W.; Parr, R. G. Development of the Colle–Salvetti Correlation-Energy Formula into a Functional of the Electron Density. *Phys. Rev. B* **1988**, *37*, 785–789. https://doi.org/10.1103/PhysRevB.37.785.

12 Stephens, P. J.; Devlin, F. J.; Chabalowski, C. F.; Frisch, M. J. Ab Initio Calculation of Vibrational Absorption and Circular Dichroism Spectra Using Density Functional Force Fields. *J. Phys. Chem.* **1994**, *98*, 11623–11627. https://doi.org/10.1021/j100096a001.

13 Grimme, S.; Ehrlich, S.; Goerigk, L. Effect of the Damping Function in Dispersion Corrected Density Functional Theory. *J. Comput. Chem.* **2011**, *32*, 1456–1465. https://doi.org/10.1002/jcc.21759.

14 Weigend, F.; Ahlrichs, R. Balanced Basis Sets of Split Valence, Triple Zeta Valence and Quadruple Zeta Valence Quality for H to Rn: Design and Assessment of Accuracy. *Phys. Chem. Chem. Phys.* **2005**, *7*, 3297–3305. https://doi.org/10.1039/b508541a.

15 Franzke, Y. J. Segmented Contracted Error-Consistent Basis Sets of Quadruple-ζ Valence Quality for One- and Two-Component Relativistic All-Electron Calculations. *J. Chem. Theory Comput.* **2023**, *19*, 1850–1861. https://doi.org/10.1021/acs.jctc.2c01244.

16 Weigend, F. Accurate Coulomb-Fitting Basis Sets for H to Rn. *Phys. Chem. Chem. Phys.* **2006**, *8*, 1057–1065. https://doi.org/10.1039/b515623h.

17 Franzke, Y. J.; Mack, F.; Weigend, F. NMR Indirect Spin–Spin Coupling Constants in a Modern Quasirelativistic Density Functional Framework. *J. Chem. Theory Comput.* **2021**, *17*, 3974–3994. https://doi.org/10.1021/acs.jctc.1c00394.

18 Rütter, D.; van Gastel, M.; Leutzsch, M.; Nöthling, N.; SantaLucia, D.; Neese, F.; Fürstner, A. Molybdenum(VI) Nitrido Complexes with Tripodal Silanolate Ligands. Structure and Electronic Character of an Unsymmetrical Dimolybdenum μ-Nitrido Complex Formed by Incomplete Nitrogen Atom Transfer. *Inorg. Chem.* **2024**, *63* (18), 8376–8389. https://doi.org/10.1021/acs.inorgchem.4c00762.

19 Wodyński, A.; Kaupp, M. Noncollinear Relativistic Two-Component X2C Calculations of Hyperfine Couplings Using Local Hybrid Functionals: Importance of the High-Density Coordinate Scaling Limit. *J. Chem. Theory Comput.* **2019**, *16* (1), 314–325. https://doi.org/10.1021/acs.jctc.9b00911.

20 Drosou, M.; Wehrung, I.; Pantazis, D. A.; Orio, M. Accurate Calculation of Electron Paramagnetic Resonance Parameters for Molybdenum Compounds. *ChemPhysChem* **2025**, *26* (22), e202500317. https://doi.org/10.1002/cphc.202500317.

21 Svenningsson, L.; Mueller, L. J.; TensorView for MATLAB: Visualizing tensors with Euler angle decoding. *Solid State Nucl. Magn. Reson.*, **2023**, *123*, 101849. https://doi.org/10.1016/j.ssnmr.2022.101849.

22 Lu, T.; Chen, F. Multiwfn: A Multifunctional Wavefunction Analyzer. *J. Comput. Chem.* **2012**, *33*, 580–592. https://doi.org/10.1002/jcc.22885.

23 Nater, D. F.; de Zwart, F. J.; Kaeffer, N.; Copéret, C. Initiating Olefin Metathesis: Alkylidenes from Molecular Mo(IV)-Oxo Species, Olefins and Base-Promoted Proton Transfer. *Chem. Sci.* **2025**, *16*, 23351–23356. https://doi.org/10.1039/D5SC06662J.

24 Bursch, M.; Neugebauer, H.; Ehlert, S.; Grimme, S. Dispersion Corrected r2SCAN Based Global Hybrid Functionals: r2SCANh, r2SCAN0, and r2SCAN50. *J. Chem. Phys.* **2022**, *156* (13), 134105. https://doi.org/10.1063/5.0086040.

25 Caldeweyher, E.; Bannwarth, C.; Grimme, S. Extension of the D3 Dispersion Coefficient Model. *J. Chem. Phys.* **2017**, *147* (3), 034112. https://doi.org/10.1063/1.4993215.

26 Bannwarth, C.; Ehlert, S.; Grimme, S. GFN2-xTB—An Accurate and Broadly Parametrized Self-Consistent Tight-Binding Quantum Chemical Method with Multipole Electrostatics and Density-Dependent Dispersion Contributions. *J. Chem. Theory Comput.* **2019**, *15* (3), 1652–1671. https://doi.org/10.1021/acs.jctc.8b01176.

27 de Souza, B. GOAT: A Global Optimization Algorithm for Molecules and Atomic Clusters. *Angew. Chem. Int. Ed.* **2025**, *64* (18), e202500393. https://doi.org/10.1002/anie.202500393.

28 Larsen, A. H.; Mortensen, J. J.; Blomqvist, J.; Castelli, I. E.; Christensen, R.; Dułak, M.; Friis, J.; Groves, M. N.; Hammer, B.; Hargus, C.; Hermes, E. D.; Jennings, P. C.; Jensen, P. B.; Kermode, J.; Kitchin, J. R.; Kolsbjerg, E. L.; Kubal, J.; Kaasbjerg, K.; Lysgaard, S.; Maronsson, J. B.; Maxson, T.; Olsen, T.; Pastewka, L.; Peterson, A.; Rostgaard, C.; Schiøtz, J.; Schütt, O.; Strange, M.; Thygesen, K. S.; Vegge, T.; Vilhelmsen, L.; Walter, M.; Zeng, Z.; Jacobsen, K. W. The Atomic Simulation Environment—A Python

Library for Working with Atoms. *J. Phys.: Condens. Matter* **2017**, *29* (27), 273002. https://doi.org/10.1088/1361-648X/aa680e.
